# Asymptotic Analysis of *q*-Recursive Sequences

**DOI:** 10.1007/s00453-022-00950-y

**Published:** 2022-05-04

**Authors:** Clemens Heuberger, Daniel Krenn, Gabriel F. Lipnik

**Affiliations:** 1 Alpen-Adria-Universität Klagenfurt, Klagenfurt, Austria; 2 Paris Lodron University of Salzburg, Salzburg, Austria; 3 Graz University of Technology, Graz, Austria

**Keywords:** Regular sequence, Recurrence relation, Digital function, Summatory function, Asymptotic analysis, Dirichlet series, Stern’s diatomic sequence, Pascal’s triangle, Thue–Morse sequence, 05A16, 11A63, 11B37, 30B50, 68Q45, 68R05, 68R15

## Abstract

For an integer $q\ge 2$, a *q*-recursive sequence is defined by recurrence relations on subsequences of indices modulo some powers of *q*. In this article, *q*-recursive sequences are studied and the asymptotic behavior of their summatory functions is analyzed. It is shown that every *q*-recursive sequence is *q*-regular in the sense of Allouche and Shallit and that a *q*-linear representation of the sequence can be computed easily by using the coefficients from the recurrence relations. Detailed asymptotic results for *q*-recursive sequences are then obtained based on a general result on the asymptotic analysis of *q*-regular sequences.

Three particular sequences are studied in detail: We discuss the asymptotic behavior of the summatory functions of

- Stern’s diatomic sequence,
- the number of non-zero elements in some generalized Pascal’s triangle and
- the number of unbordered factors in the Thue–Morse sequence.

For the first two sequences, our analysis even leads to precise formulæ without error terms.

## Introduction

*q*-**Recursive Sequences**

We study a special class of recursively defined sequences, the so-called *q*-*recursive sequences*. Here *q* is an integer and at least 2, and *q*-recursive sequences are sequences which satisfy a specific type of recurrence relation: Roughly speaking, every subsequence whose indices run through a residue class modulo $q^M$ is a linear combination of subsequences where for each of these subsequences, the indices run through a residue class modulo $q^m$ for some $m < M$.

It turns out that this property is quite natural and many combinatorial sequences are in fact *q*-recursive. A simple nontrivial example of such a sequence^1^ is when *h*(*n*) is the largest power of 2 less than or equal to *n*; see [36,  A053644]. Then we have $h(2n)=2 h(n)$ and $h(2n+1)=2 h(n)$ for $n\ge 1$ as well as $h(1)=1$, so clearly $q=2$, and we set $M=1$ and $m=0$. This is because the left-hand sides of the two recurrence relations contain $2^1n$ shifted by 0 and 1, and because the right-hand sides only contain $2^0n$ (and no shifts). Another example are divide-and-conquer recurrences, see [24,  Equation (1.2)].

*q*-**Regular Sequences**

The concept of *q*-recursive sequences is related to *q*-regular sequences introduced by Allouche and Shallit [1]. One definition of a *q*-regular sequence *x* is that every subsequence of the form $x(q^jn+r)$ can be written as a linear combination of the same finite number of sequences; see Sect. 2 for more details and a precise description. Again the sequence *h* [36,  A053644] is an example, now for a 2-regular sequence; it satisfies $h(2^jn+r)=2^j h(n)$ for^2^$n \ge 1$, $j\ge 0$ and $0\le r<2^j$, so every $h(2^jn+r)$ can be written in terms of *h*(*n*).

Equivalently, every *q*-regular sequence can be modeled by a *q*-linear representation. Here *x*(*n*) is one component of a vector *v*(*n*), and there exist matrices $A_r$ with $v(qn+r)=A_r v(n)$ for all $0 \le r < q$ and $n \ge 0$; see also Sect. 2.

**Linear Representation of** *q*-**Recursive Sequences.**

One main result of this paper is that every *q*-recursive sequence is indeed *q*-regular; see Sect. 3.2. Even more can be said: Theorem A provides an explicit *q*-linear representation of the sequence.

In Sect. 3.3, we significantly improve our results for a special case of *q*-recursive sequences, namely where $M=m+1$.

**Asymptotics of ***q*-**Recursive Sequences.**

After exploring the concept of *q*-recursive sequences itself, we investigate the asymptotic behavior of the summatory functions of *q*-recursive sequences, i.e., sequences of partial sums. There exist explicit results for many particular regular sequences and also some quite general results. Dumas [13] as well as the first two authors of this paper together with Prodinger [20, 22] studied the asymptotics of *q*-regular sequences in general. The two works [20, 22] will also be one of the main ingredients for obtaining the asymptotic behavior of *q*-recursive sequences. We present details in Sect. 4. We investigate an important special case where the asymptotic behavior can be directly determined from the *q*-recursive sequence without constructing the representation as a *q*-regular sequence.

**Explicit Precise Asymptotics for Three Particular Sequences.**

We also investigate three specific *q*-recursive sequences in-depth. In particular, we derive asymptotic results for their summatory functions as well as explain and illustrate the connection between these results and the fact that the sequences are *q*-recursive. To be more specific, we analyze

- Stern’s diatomic sequence in Sect. 5,
- the number of non-zero entries in a generalized Pascal’s triangle in Sect. 6, and
- the number of unbordered factors in the Thue–Morse sequence in Sect. 7.

For the first two sequences, our analysis even leads to precise formulæ without error terms.

**Proofs.**

We finally complete this paper by giving proofs of our results; these are collected in Sect. 8.

## Brief Introduction to *q*-Regular Sequences

The concept of *q*-regular sequences^3^ was first introduced by Allouche and Shallit [1] in 1992, and a lot of research on them has been done since then; see for example Allouche and Shallit [2] and [3], Bell [4], and Coons and Spiegelhofer [11].

The parameter *q* acts as a base (or radix); therefore the term *digital function* arises in context of such sequences. We start by giving a definition; see Allouche and Shallit [2].

Let $q\ge 2$ be a fixed integer and $x:{\mathbb {N}}_{0}\rightarrow {\mathbb {C}}$ be a sequence^4^. Then *x* is called *q*-*regular* if the complex vector space generated by its *q*-kernelhas finite dimension. In other words, a sequence *x* is *q*-*regular* if there are $\Delta \in {\mathbb {N}}_{0}$ and sequences $x_{1}$, $\dots$, $x_{\Delta }$ such that for every $j\in {\mathbb {N}}_{0}$ and $0\le r < q^{j}$ there exist $c_{1}$, $\dots$, $c_{\Delta }\in {\mathbb {C}}$ with

$$\begin{aligned} x(q^{j}n + r) = \sum _{i=1}^{\Delta }c_{i}x_{i}(n) \end{aligned}$$

for all $$n \ge 0$$.

By Allouche and Shallit [1,  Theorem 2.2], a complex-valued sequence *x* is *q*-regular if and only if there exist a vector-valued sequence $v:{\mathbb {N}}_{0}\rightarrow {\mathbb {C}}^{D}$ for some $D\in {\mathbb {N}}$ whose first component coincides with *x* and matrices $A_{0}$, ..., $A_{q-1}$ such that

$$\begin{aligned} v(qn + r) = A_{r}v(n) \end{aligned}$$

holds for all $0\le r < q$ and $n \ge 0$. If this is the case, the tuple $(A_{0},\dots ,A_{q-1},v)$ is called *q*-*linear representation* of *x*, and *D* is said to be its *dimension*.

At this point, we note that a *q*-linear representation $(A_{0}, \dots , A_{q-1},v)$ of a sequence *x* immediately leads to an explicit expression for *x*(*n*) by induction: Let $d_{L-1}\ldots d_{0}$ be the *q*-ary digit expansion of *n*. Then we have

2.1 $$\begin{aligned} x(n) = e_{1}A_{d_{0}}\ldots A_{d_{L-1}}v(0), \end{aligned}$$

where $e_{1} = (1, 0, \ldots , 0)$. In particular, the *n*th element *x*(*n*) can be computed in $O(\log n)$ operations in ${\mathbb {C}}$.

The prototypical and probably best-known example of a *q*-regular sequence is the binary sum of digits.

### Example 2.1

(Binary Sum of Digits [36,  A064547]) For $n\in {\mathbb {N}}_{0}$, let *s*(*n*) denote the number of ones in the binary expansion of *n*. Then we clearly have

2.2 $$\begin{aligned} s(2n) = s(n)\text { and } s(2n + 1) = s(n) + 1 \end{aligned}$$

for all $n \ge 0$ and $s(0)=0$. By induction we obtain

$$\begin{aligned} s(2^{j}n + r) = s(n) + s(r)\cdot 1 \end{aligned}$$

for all $n \ge 0$, $j \ge 0$ and $0\le r < 2^{j}$. This means that the complex vector space generated by the kernel ${\mathcal {K}}_{q}(s)$ is also generated by *s* and $n\mapsto 1$ and thus, the sequences *s* is 2-regular.

A 2-linear representation $(A_{0}, A_{1}, v)$ of *s* is given by

$$\begin{aligned} A_{0} = \begin{pmatrix} 1 &{}\quad 0\\ 0 &{}\quad 1 \end{pmatrix},\quad A_{1} = \begin{pmatrix} 1 &{}\quad 1\\ 0 &{}\quad 1 \end{pmatrix} \text { and } v = \begin{pmatrix} s\\ n\mapsto 1 \end{pmatrix}, \end{aligned}$$

where the corresponding recurrence relations $v(2n) = A_{0}v(n)$ as well as $v(2n + 1) = A_{1}v(n)$ can be checked easily by using (2.2).

## *q*-Recursive Sequences

In this section, we introduce the concept of *q*-recursive sequences, investigate how linear representations of these sequences look like and thereby conclude that these sequences are *q*-regular. We will also investigate a more restricted set-up. This special case (as we will call it from now on in contrast to the general case) is important; two instances will be discussed in Sects. 6 and 7.

### Definitions

We start by giving the definition of our sequences of interest.

#### Definition 3.1

(*q*-*Recursive Sequence*) Let $q\ge 2$, $M > m \ge 0$, $\ell \le u$ and  be fixed integers. Let *x* be a sequence.

If there are constants $c_{s,k}\in {\mathbb {C}}$ for all $0\le s < q^{M}$ and $\ell \le k\le u$ such that3.1holds for all $n\ge n_{0}$ and $0\le s<q^{M}$, then we say that the sequence *x* is *q*-*recursive with offset *$n_{0}$, *exponents* *M* *and* *m*, *index shift bounds* $\ell$ *and* *u*, *and coefficients* $(c_{s,k})_{0\le s<q^M,\ell \le k\le u}$.

We use the convention that if any of the parameters *q*, $n_{0}$, *M*, *m*, $\ell$, *u*, $(c_{s,k})_{0\le s<q^M,\ell \le k\le u}$ is not mentioned for a recursive sequence, then we assume that a value of this parameter exists such that the sequence is recursive with this value of the parameter.

The sequence where *h*(*n*) is the largest power of 2 less than or equal to *n* [36, A053644], that was mentioned in the introduction, is indeed a 2-recursive sequence with offset 1, as $h(2n)=2 h(n)$ and $h(2n+1)=2 h(n)$ hold for $n\ge 1$. On the other hand, the binary sum of digits as introduced in Example 2.1 does not directly^5^ fit into this framework, because the constant sequence appears on the right-hand side; see (2.2). For a discussion of such inhomogeneous *q*-recursive sequences we refer to Corollary D.

Before considering a slightly more involved example, we clarify the role of the restriction on $n_0$.

#### Remark 3.2

The condition  in Definition 3.1 is necessary because for $n=n_0$, (3.1) reduces toand so the smallest argument of *x* on the right-hand side is $q^{m}n_{0} + \ell$, which is non-negative by the given condition and therefore indeed a valid argument.

**Table 1:** First few elements of *p*

*n*	0	1	2	3	4	5	6	7	8	9	10
*p*(*n*)	0	1	3	5	9	11	15	19	27	29	33

#### Example 3.3

(Odd Entries in Pascal’s Triangle [36,   A006046]) Let *p*(*n*) be the number of odd entries in the first *n* rows of Pascal’s triangle. The first few elements are given in Table 1.

By Lucas’ theorem on binomial coefficients modulo a prime, the number of odd entries in row *n* of Pascal’s triangle is given by $2^{s(n)}$, where *s*(*n*) is the binary sum of digits of *n*; see also Fine [15,  Theorem 2]. This implies thatas well ashold for all $n \ge 0$. Thus, the sequence *p* is 2-recursive with exponents $M=1$ and $m=0$, index shift bounds $\ell =0$ and $u=1$, and offset $n_{0}=0$.

From Allouche and Shallit [1,  Example 14 and Theorem 3.1], we know that the sequence *p* is 2-regular as well. This is no coincidence: In the following, we will show that each *q*-recursive sequence is *q*-regular. Furthermore, if the recurrence relations in (3.1) are known, we can even give an explicit *q*-linear representation of *x*.

In analogy to Definition 3.1, we also introduce *q*-*regular sequences with offset*.

#### Definition 3.4

(*q*-*Regular Sequence with Offset*) Let $q \ge 2$ and $n_0 \ge 0$ be fixed integers. A sequence *x* is said to be *q*-*regular with offset* $n_{0}$ if there exist a vector-valued sequence $v:{\mathbb {N}}_{0}\rightarrow {\mathbb {C}}^{D}$ for some $D\in {\mathbb {N}}$ whose first component coincides with *x* and matrices $A_{0}$, ..., $A_{q-1}$ such that $v(qn + r) = A_{r}v(n)$ holds for all $0\le r < q$ and $n \ge n_{0}$. In this case, we say that $(A_{0},\ldots , A_{q-1}, v)$ is a *q*-*linear representation with offset* $n_{0}$ of *x*.

#### Remark 3.5

A *q*-regular sequence with offset 0 is *q*-regular in the usual sense. Likewise, every *q*-linear representation with offset 0 is a *q*-linear representation as introduced in Sect. 2.

### Reduction to *q*-Regular Sequences in the General Case

It turns out that every *q*-recursive sequence with any offset is indeed *q*-regular (Corollary C). This is an implication of the following two results:

- Theorem A explicitly constructs a *q*-linear representation with offset $n_{1}$ of *q*-recursive sequences with offset $n_{0}$, where $n_{1}\in {\mathbb {N}}$ is explicitly given. This means that such sequences are *q*-regular with offset $n_{1}$.
- Theorem B states that every *q*-regular sequence with some offset is *q*-regular (without offset) as well. Also here, an explicit *q*-linear representation of *x* is given.

#### Theorem A

Let *x* be a *q*-recursive sequence with offset $n_{0}$, exponents *M* and *m* and index shift bounds $\ell$ and *u*. Furthermore, set^6^

3.2a $$\begin{aligned} \ell '&{{:=}} {\left\lfloor \frac{(\ell +1) q^{M-m} - q^{M}}{q^{M-m}-1}\right\rfloor } {\llbracket \ell < 0 \rrbracket } \end{aligned}$$

and

3.2b $$\begin{aligned} u'&{{:=}} q^{m} - 1 + {\left\lceil \frac{uq^{M-m}}{q^{M-m}-1}\right\rceil } {\llbracket u > 0 \rrbracket }. \end{aligned}$$

Then *x* is *q*-regular with offset $n_{1} = n_{0} - {\left\lfloor \ell ' /q^{M}\right\rfloor }$, and a *q*-linear representation $(A_{0}, \dots , A_{q-1}, v)$ with offset $n_{1}$ of *x* is given as follows:

- The vector-valued sequence *v* is given in block form by
  3.3 $$\begin{aligned} v = \begin{pmatrix} v_{0}\\ \vdots \\ v_{M-1} \end{pmatrix}, \end{aligned}$$
  where the blocks are of the following form: For $0\le j < m$, the block $v_{j}$ has the form
  3.4a $$\begin{aligned} v_{j} = \begin{pmatrix} x\circ (n\mapsto q^{j}n)\\ \vdots \\ x\circ (n\mapsto q^{j}n + q^{j} - 1) \end{pmatrix}, \end{aligned}$$
  and for $m\le j < M$, the block $v_{j}$ has the form^7^
  3.4b $$\begin{aligned} v_{j} = \begin{pmatrix} x\circ (n\mapsto q^{j}n + \ell ')\\ \vdots \\ x\circ (n\mapsto q^{j}n + q^{j} - q^{m} + u') \end{pmatrix}. \end{aligned}$$
- The matrices $A_{0}$, ..., $A_{q-1}$ of the *q*-linear representation with offset $n_{1}$ can be computed by using the coefficients in (3.1); an explicit formula for the rows of these matrices is given in (8.8).

The linear representation $(A_{0}, \ldots , A_{q-1}, v)$ does not depend on the offset $n_{0}$.

#### Remark 3.6

1. We easily verify that $\ell '\le 0$ holds and it is clear that $u'\ge q^m-1 \ge 0$. Thus $\ell ' \le u'$. This implies that the blocks $v_j$ for $m\le j<M$ in (3.4b) are indeed non-empty.
2. It is easy to check that *x* itself is a component of *v*. For $m=0$, this is due to the fact that we have $\ell '\le 0 \le u'$. However, it can happen that *x* is not the *first component* of *v* (as it is required for a linear representation). Then a simple permutation of the components of *v* brings *x* to its first component.
3. The dimension of the *q*-linear representation is
  $$\begin{aligned} \frac{q^{M} - 1}{q - 1} + (M - m)\bigl (u' - \ell ' - q^{m} + 1\bigr ), \end{aligned}$$
  which is possibly very big. However, we can always apply a minimization algorithm in order to decrease the dimension of the linear representation as far as possible. Such an algorithm is presented in Berstel and Reutenauer [5,  Chapter 2] for recognizable series, but can be applied on regular sequences as well; see [21]. SageMath [37] provides an implementation of this minimization algorithm.
4. The statement of Theorem A for $M = 1$ and $m = n_{0} = 0$ is mentioned by the first two authors of this article in [20,  Remark 5.1]. $\square$

In order to put the main aspects of the previous result across, we present two examples: The first one is a simple continuation of Example 3.3, and the second one discusses a *q*-recursive sequence with more involved parameters. While the latter might not seem to be very natural, it is an intentionally made choice to keep things illustrative and comprehensible. For further natural combinatorial examples we refer to Sects. 5, 6 and 7.

#### Example 3.7

(Odd Entries in Pascal’s Triangle, continued) Let *p*(*n*) again be the number of odd entries in the first *n* rows of Pascal’s triangle. As already mentioned (Example 3.3), *p* is 2-recursive with exponents $M=1$ and $m=0$ and index shift bounds $\ell =0$ and $u=1$ as well as3.5for all $n \ge 0$. Due to Theorem A, *p* is also 2-regular (with offset $n_{1} = 0$) and a 2-regular representation of *p* can be found as follows. We have $\ell ' = 0$ and $u' = 2$, and it is due to the relation $m = M - 1 = 0$ that the vector *v* only consists of one block, namely

$$\begin{aligned} v = v_{M-1} = v_{0} = \begin{pmatrix} p\\ p\circ (n\mapsto n + 1)\\ p\circ (n\mapsto n + 2) \end{pmatrix}. \end{aligned}$$

Moreover, we can determine the matrices $A_{0}$ and $A_{1}$ in various ways: By (8.8), these matrices areHowever, these matrices can also be obtained in an ad hoc fashion, namely by inserting 2*n* and $2n + 1$ into *v* and then component-wise applying (3.5). For example, let us take a look at the third row of $A_{0}$: We have to consider the third component of *v*, which is $p\circ (n\mapsto n+2)$. We insert 2*n*, which results in $p\circ (n\mapsto 2n+2)$, and we obtainby (3.5). Thus, we have a 3 in the second column, because $p\circ (n\mapsto n + 1)$ is the second component of *v*, and a 0 in the third column, because $p\circ (n\mapsto n + 2)$ is the third component of *v*. Generally speaking, the rows of $A_{r}$ that correspond to the last block $v_{M-1}$ always consist of shifted copies of the coefficients in the recurrence relations.

The “step” between the second row and the third row of $A_{0}$ and between the first row and the second row of $A_{1}$ is caused by the following fact: After inserting 2*n* or $2n + 1$, it can happen that the remainder is too large to apply the given recurrence relations directly. For instance, this was the case when determining the third row of $A_{0}$ above: After inserting 2*n*, we have obtained $p\circ (n\mapsto 2n+2)$, and we had to rewrite this to $p\circ (n\mapsto 2(n+1))$ to be able to apply (3.5). This increase of the argument by 1 causes the shift of the entries in the matrix to the right by 1. For a more detailed description of this effect, we refer to the two different cases in Part 3 of the proof of Theorem A in Sect. 8.1 and to (8.8).

Note that the dimension of this linear representation is not minimal since the sequence $p\circ (n\mapsto n+2)$ can be omitted. This is due to the following two facts: The third columns of $A_{0}$ and $A_{1}$ correspond to $p\circ (n\mapsto n+2)$. All non-zero elements of these columns are in the last row, which again corresponds to $p\circ (n\mapsto n+2)$. This reduction is possible because the coefficient of $p(n+1)$ in the left recurrence relation of (3.5) is zero.

#### Example 3.8

Consider the 2-recursive sequence *x* with exponents $M = 3$ and $m = 1$ given by the recurrence relations

3.6 $$\begin{aligned} \begin{aligned} x(8n)&= -1x(2n - 1) + 0x(2n) + 1x(2n + 1),\\ x(8n + 1)&= -11x(2n - 1) + 10x(2n) + 11x(2n + 1),\\ x(8n + 2)&= -21x(2n - 1) + 20x(2n) + 21x(2n + 1),\\ x(8n + 3)&= -31x(2n - 1) + 30x(2n) + 31x(2n + 1),\\ x(8n + 4)&= -41x(2n - 1) + 40x(2n) + 41x(2n + 1),\\ x(8n + 5)&= -51x(2n - 1) + 50x(2n) + 51x(2n + 1),\\ x(8n + 6)&= -61x(2n - 1) + 60x(2n) + 61x(2n + 1),\\ x(8n + 7)&= -71x(2n - 1) + 70x(2n) + 71x(2n + 1) \end{aligned} \end{aligned}$$

for all $n \ge 0$. So for the sake of recognition, the coefficients $(c_{s,k})_{0\le s<8, -1\le k\le 1}$ are given by $c_{s,k} = (-1)^{{\llbracket k < 0 \rrbracket }}10s + k$. The index shift bounds of *x* are $\ell = -1$ and $u = 1$, and its offset is $n_{0} = 0$. With the notation of Theorem A, we further find $\ell ' = -3$ and $u' = 3$.

Due to Theorem A, *x* is 2-regular with offset $n_{1} = 1$, and by (3.4) and (8.8), a 2-linear representation with offset $n_{1} = 1$ of *x* is given by $(A_{0}, A_{1}, v)$ withas well asAgain, the matrices can also be obtained ad hoc, by inserting 2*n* and $2n + 1$ into the components and, if needed, component-wise applying the relations of (3.6). For example, the fourth row of $A_{1}$ corresponds to $x\circ (n\mapsto 2n-1)$, i.e., the fourth component of *v*. Inserting $2n + 1$ yields $x\circ (n\mapsto 2(2n + 1) -1)= x\circ (n\mapsto 4n + 1)$, which itself is the 13th component of *v*. Thus, we have a 1 in the 13th column in the fourth row of $A_{1}$.

The “interesting” part of the matrices $A_{0}$ and $A_{1}$ is given by entries in rows corresponding to $v_{M-1} = v_{2}$ and columns corresponding to $v_{m} = v_{1}$. It is marked by the green and red boxes, respectively, and the entries can be obtained exactly as described in the previous example. Here the application of (3.6) is indeed needed and again leads to a block of shifted copies of the coefficients in the recurrence relations. Also here, one can see the “steps” in the matrices that were described in Example 3.7.

Up to now, we have reduced *q*-recursive sequences to *q*-regular sequences with some offset. Next, we get rid of this offset; Allouche and Shallit implicitly do such an offset correction for offset 1 in the proof of [1,  Lemma 4.1].

#### Theorem B

Let *x* be a *q*-regular sequence with offset $n_{0}$, and let $(A_{0},\ldots ,A_{q-1}, v)$ be a *q*-linear representation with offset $n_{0}$ of *x*. Then *x* is *q*-regular and a *q*-linear representation $({\widetilde{A}}_{0}, \dots , {\widetilde{A}}_{q-1}, {\widetilde{v}})$ of *x* is given as follows:

- The vector-valued sequence ${\widetilde{v}}$ is given in block form by
  3.7 $$\begin{aligned} {\widetilde{v}} = \begin{pmatrix} v\\ \delta _{0}\\ \vdots \\ \delta _{n_{0}-1} \end{pmatrix}, \end{aligned}$$
  where $\delta _{k}:{\mathbb {N}}_{0}\rightarrow {\mathbb {C}}$ is defined by $\delta _{k}(n) = {\llbracket n = k \rrbracket }$ for all $0\le k < n_{0}$ and $n\ge 0$.
- Let $D\in {\mathbb {N}}$ be the dimension of *v*. Moreover, for $0\le r < q$ and $0 \le k < n_{0}$, let^8^$w_{r,k} {{:=}} v(qk + r) - A_{r}v(k)\in {\mathbb {C}}^{D}$, and let $W_{r}$ be the $D\times n_{0}$ matrix which has columns $w_{r,0}$, ..., $w_{r,n_{0}-1}$. Then for all $0\le r < q$, the matrix ${\widetilde{A}}_{r}$ is given in block form by
  3.8 $$\begin{aligned} {\widetilde{A}}_{r} = \begin{pmatrix} A_{r} &{} W_{r}\\ 0 &{} J_{r} \end{pmatrix}, \end{aligned}$$
  where  is the matrix defined by
  3.9 $$\begin{aligned} J_{r} {{:=}} \bigl ({\llbracket jq = k - r \rrbracket }\bigr )_{\begin{array}{c} 0\le k< n_{0}\\ 0\le j < n_{0} \end{array}}. \end{aligned}$$
  The matrix $J_r$ is a lower triangular matrix with diagonal
  $$\begin{aligned} \text { diag}(J_{r}) = \bigl ({\llbracket r = 0 \rrbracket }, 0, \dots , 0\bigr ). \end{aligned}$$

#### Corollary C

Every *q*-recursive sequence *x* with any offset is *q*-regular and a *q*-linear representation of *x* is given as the combination of the explicit constructions of the *q*-linear representations from Theorem A and Theorem B.

While Sect. 3 up to this point (in particular Definition 3.1) considered homogeneous recursive sequences, also inhomogeneities can occur. An example is, as already mentioned, the binary sum of digits, where the constant sequence appears. In the following corollary, we deal with such inhomogeneous recursive sequences.

#### Corollary D

Let $q\ge 2$, $M > m \ge 0$, $\ell \le u$ and  be fixed integers. Furthermore, let *x* be a sequence such that for all $0\le s < q^{M}$ there exist *q*-regular sequences $g_{s}$ and constants $c_{s,k}\in {\mathbb {C}}$ for $\ell \le k \le u$ with3.10for all $n\ge n_{0}$.

Then *x* is *q*-regular and a *q*-linear representation of *x* can be constructed straightforwardly by combining the explicit constructions of the *q*-linear representations from Theorem A and Theorem B with *q*-linear representations of shifted versions of the sequences $g_{s}$.

#### Remark 3.9

The construction of a *q*-linear representation of a *q*-recursive sequence (given by recurrence relations as in (3.1) or in (3.10)) with offset has been included [26] in SageMath [37].

### Reduction to *q*-Regular Sequences in a Special Case

We now study a specific case of *q*-recursive sequences, namely *q*-recursive sequences with exponents $M=m+1$ and *m* and index shift bounds $\ell =0$ and $u=q^{m}-1$ for some $m\in {\mathbb {N}}_{0}$. The study of this case is well-motivated: First of all, it will turn out in Sects. 6 and 7 that this choice of parameters is quite natural, i.e., we will see examples where subsequences of indices modulo $q^{m+1}$ equal linear combinations of subsequences of indices modulo $q^{m}$. Moreover, we can give the matrices of the linear representation in a simpler form than in Theorem A, and the upper bound $u'$ can be improved significantly. Finally, we show that the asymptotics of the summatory functions of this special case of sequences can be obtained directly from the recurrence relations in (3.1), without knowing a linear representation of the sequence explicitly.

Note that in this section we assume the offset to be $n_{0} = 0$, mainly for the sake of readability. However, we want to emphasize that all results can be stated for arbitrary offset $n_{0}\in {\mathbb {N}}_{0}$ as well, using Theorem B.

We start by giving an analogon of Theorem A for our special case.

#### Theorem E

Let *x* be a *q*-recursive sequence with exponents $M=m+1$ and *m* and index shift bounds $\ell =0$ and $u=q^{m} - 1$ and coefficients $(c_{s,k})_{0\le s< q^{m + 1}, 0\le k < q^m}$. We define the matrices

3.11 $$\begin{aligned} B_{r} = (c_{rq^m+d,k})_{\begin{array}{c} 0 \le d< r q^{m} \\ 0\le k < q^{m} \end{array}} \end{aligned}$$

for $0 \le r < q$. Then *x* is *q*-regular and a *q*-linear representation $(A_{0}, \dots , A_{q-1}, v)$ of *x* is given as follows:

- The vector-valued sequence *v* is given in block form by
  3.12 $$\begin{aligned} v = \begin{pmatrix} v_{0}\\ \vdots \\ v_{m} \end{pmatrix}, \end{aligned}$$
  where for $0\le j \le m$, the block $v_{j}$ has the form
  3.13 $$\begin{aligned} v_{j} = \begin{pmatrix} x\circ (n\mapsto q^{j}n)\\ \vdots \\ x\circ (n\mapsto q^{j}n + q^{j} - 1) \end{pmatrix}. \end{aligned}$$
- For $0 \le r < q$, the matrices $A_{r}$ are given in block form by
  3.14 $$\begin{aligned} A_{r} = \begin{pmatrix} J_{r0} &{}\quad J_{r1}\\ 0 &{}\quad B_{r} \end{pmatrix}, \end{aligned}$$
  where  and . Furthermore, for $0\le r < q$, the matrices $J_{r0}$ are upper triangular matrices with zeros on the diagonal, and the matrices $J_{r0}$ and $J_{r1}$ are given explicitly by the first case of (8.8) (with $u'$ replaced by $q^m-1$).

#### Remark 3.10

1. The structure of *v* is the same as in Theorem A. In particular, the blocks $v_{j}$ with $0\le j< m$ coincide with the blocks $v_{j}$ from Theorem A given in (3.4a).
2. The matrices $J_{r0}$ and $J_{r1}$ can be decomposed into blocks of identity matrices and zero matrices of smaller dimensions, which are horizontally shifted depending on *r*. For an illustration we refer to Example 3.11.
3. The last component of *v* is $x\circ (n\mapsto q^mn+q^m-1)$ in contrast to $x\circ (n\mapsto q^mn+u')$ when using Theorem A. This means that using Theorem E leads to a linear representation whose dimension is $\frac{q^{m+1} - q}{q-1}$ less than the dimension achieved by Theorem A.
4. In the case $m = 0$, only rather special sequences can be handled by Theorem E. For instance, for $q=2$ and $m=0$, only sequences of the form $x(n) = x(0)a^{s(n)}$, where *s*(*n*) is the binary sum of digits of *n* and *a* is some constant, fulfill the assumptions of this theorem. For all other *q*-recursive sequences with $m = 0$, Theorem A has to be used. $\square$

The following example will allow us to illustrate Theorem E. For the sake of simplicity, we again choose an artificial example.

#### Example 3.11

Let us study the 2-recursive sequence *x* with exponents $M=3$ and $m=2$ given by the recurrence relations

$$\begin{aligned} f(8n)&= f(4n) + f(4n+1) + f(4n+2) + f(4n+3),\\ f(8n + 1)&= f(4n) + f(4n+1) + f(4n+2) + f(4n+3),\\ f(8n + 2)&= f(4n) + f(4n+1) + f(4n+2) + f(4n+3),\\ f(8n + 3)&= f(4n) + f(4n+1) + f(4n+2) + f(4n+3),\\ f(8n + 4)&= 2f(4n) + 2f(4n+1) + 2f(4n+2) + 2f(4n+3),\\ f(8n + 5)&= 2f(4n) + 2f(4n+1) + 2f(4n+2) + 2f(4n+3),\\ f(8n + 6)&= 2f(4n) + 2f(4n+1) + 2f(4n+2) + 2f(4n+3),\\ f(8n + 7)&= 2f(4n) + 2f(4n+1) + 2f(4n+2) + 2f(4n+3) \end{aligned}$$

for all $n \ge 0$. Then we haveBy Theorem E, *x* is 2-regular and a 2-linear representation $(A_{0}, A_{1}, v)$ of *x* is given by

$$\begin{aligned} v = \begin{pmatrix} x\\ x\circ (n\mapsto 2n)\\ x\circ (n\mapsto 2n + 1)\\ x\circ (n\mapsto 4n)\\ x\circ (n\mapsto 4n+1)\\ x\circ (n\mapsto 4n+2)\\ x\circ (n\mapsto 4n+3) \end{pmatrix} \end{aligned}$$

as well asThe dark gray boxes mark the matrices $J_{r0}$ and $J_{r1}$, whereas the smaller, light gray boxes mark the shifted identity matrices mentioned in Remark 3.10.

## Asymptotics

We want to study the asymptotic behavior for *q*-recursive sequences (or, to be precise, of their summatory functions). As we have already seen that such sequences are *q*-regular, we can apply the results of [20]. This is indeed what we do, however, our set-up here is more specific than *q*-regular sequences in general, because the sequences are given by particular recurrence relations. This leads to more specific results here.

We start by briefly discussing the growth of matrix products, in particular in conjunction with the joint spectral radius. This is one important quantity determining the asymptotics of a sequence. Beside that, the eigenvalues of the sum of matrices of a *q*-linear representation play an important role.

Again, we will distinguish between the general case and the special case introduced in Sect. 3.3.

### Growth of Matrix Products

Before presenting previous results and adapting them to our purposes, we recall the notion of the joint spectral radius and introduce some related notions.

We fix a vector norm $\Vert {\,\cdot \,} \Vert$ on ${\mathbb {C}}^{D}$ and consider its induced matrix norm.

#### Definition 4.1

Let ${\mathcal {G}}$ be a finite set of $D\times D$ matrices over ${\mathbb {C}}$.

- The joint spectral radius of ${\mathcal {G}}$ is defined as
  $$\begin{aligned} \rho ({\mathcal {G}}){{:=}} \lim _{k\rightarrow \infty } \sup \{\Vert {G_{1}\ldots G_{k}} \Vert ^{1/k}\mid G_1,\ldots , G_k\in {\mathcal {G}}\}. \end{aligned}$$
- We say that ${\mathcal {G}}$ has the *finiteness property* if there exists a $k\in {\mathbb {N}}$ such that
  $$\begin{aligned} \rho ({\mathcal {G}})=\sup \{\Vert {G_{1}\ldots G_{k}} \Vert ^{1/k}\mid G_1,\ldots , G_k\in {\mathcal {G}}\}. \end{aligned}$$
- We say that ${\mathcal {G}}$ has the *simple growth property* if
  $$\begin{aligned} \Vert {G_1\ldots G_k} \Vert =O(\rho ({\mathcal {G}})^k) \end{aligned}$$
  holds for all $G_1$, ..., $G_k\in {\mathcal {G}}$ and $k\rightarrow \infty$.

#### Remark 4.2

1. In the definition of the joint spectral radius, the limit can be replaced by an infimum over all $k \ge 1$; see Rota and Strang [34] and also [20,  Section 7.2]. In particular, the limit in the definition of the joint spectral radius always exists.
2. As any two norms on ${\mathbb {C}}^{D\times D}$ are equivalent, the definitions of the joint spectral radius and the simple growth property do not depend on the chosen norm. The finiteness property, however, depends on the chosen norm; see Remark 7.4 for an example.
3. The finiteness property implies the simple growth property; see [20,  Section 7.2].
4. The set
  $$\begin{aligned} {\mathcal {G}}{{:=}} \left\{ \begin{pmatrix}1&{}\quad 1\\ 0&{}\quad 1\end{pmatrix}\right\} \end{aligned}$$
  has joint spectral radius 1, but not the simple growth property, because the *k*th power of the only matrix in ${\mathcal {G}}$ equals
  $$\begin{aligned} \begin{pmatrix}1&{}\quad 1\\ 0&{}\quad 1\end{pmatrix}^k = \begin{pmatrix}1&{}\quad k\\ 0&{}\quad 1\end{pmatrix}. \end{aligned}$$

$$\square $$

In Lemma 4.5, we will study sufficient conditions under which sets of block triangular matrices have the simple growth property.

### Asymptotics for Regular Sequences

In order to obtain the asymptotics for the summatory function of *q*-recursive sequences, we now apply a result of the first two authors of this article on the asymptotic behavior of *q*-regular sequences [20, Theorem A]. So let *x* be a *q*-recursive sequence with *q*-linear representation $(A_{0}, \dots , A_{q-1}, v)$, and setFor a square matrix *G*, let $\sigma (G)$ denote the set of eigenvalues of *G* and by $m_{G}(\lambda )$ the size of the largest Jordan block of *G* associated with some $\lambda \in {\mathbb {C}}$. In particular, we have $m_{G}(\lambda ) = 0$ if $\lambda \notin \sigma (G)$.

Then we choose $R > 0$ as follows: If the set  has the simple growth property, then we set $R=\rho ({\mathcal {A}})$. Otherwise, we choose $R > \rho ({\mathcal {A}})$ such that there is no eigenvalue $\lambda \in \sigma (C)$ with $\rho ({\mathcal {A}}) < |\lambda |\le R$. Furthermore, we let

$$\begin{aligned} {\mathcal {X}}(s) = \sum _{n\ge 1}n^{-s}x(n) \text { and} {\mathcal {V}}(s) = \sum _{n\ge 1}n^{-s}v(n) \end{aligned}$$

denote the Dirichlet series corresponding to *x* and *v*. Now we are ready to state the result.

#### Theorem F

(Asymptotic Analysis of *q*-Regular Sequences  [20,  Theorem A]) With the notations above, we have^9^4.1as $N\rightarrow \infty$, where $\Phi _{\lambda k}$ are suitable 1-periodic functions. If there are no eigenvalues $\lambda \in \sigma (C)$ with $|\lambda | \le R$, the *O*-term can be omitted.

For $|\lambda | > R$ and $0\le k < m_{C}(\lambda )$, the function $\Phi _{\lambda k}$ is Hölder continuous with any exponent smaller than $\log _{q}(|\lambda |/R)$.

The Dirichlet series ${\mathcal {V}}(s)$ converges absolutely and uniformly on compact subsets of the half plane ${\text {Re}}s > \log _{q}R + 1$ and can be continued to a meromorphic function on the half plane ${\text {Re}}s > \log _{q}R$. It satisfies the functional equation4.2for ${\text {Re}}s > \log _{q}R$. The right-hand side of (4.2) converges absolutely and uniformly on compact subsets of ${\text {Re}}s > \log _{q}R$. In particular, ${\mathcal {V}}(s)$ can only have poles where $q^{s}\in \sigma (C)$.

For $\lambda \in \sigma (C)$ with $|\lambda | > R$ and $0\le k<m_{C}(\lambda )$, the Fourier series

4.3 $$\begin{aligned} \Phi _{\lambda k}(u) = \sum _{\mu \in {\mathbb {Z}}}\varphi _{\lambda k\mu }\exp (2\mu \pi iu) \end{aligned}$$

converges pointwise for $u\in {\mathbb {R}}$ where the Fourier coefficients $\varphi _{\lambda k\mu }$ are given by the singular expansion

4.4 $$\begin{aligned} \frac{x(0) + {\mathcal {X}}(s)}{s} \asymp \sum _{\begin{array}{c} \lambda \in \sigma (C)\\ |\lambda | > R \end{array}} \; \sum _{\mu \in {\mathbb {Z}}}\; \sum _{0\le k< m_C(\lambda )} \frac{\varphi _{\lambda k\mu }}{\bigl (s - \log _{q}\lambda - \frac{2\mu \pi i}{\log q}\bigr )^{k+1}} \end{aligned}$$

for $${\text {Re}}s > \log _{q}R$$.

#### Remark 4.3

1. [20,  Theorem A] only uses the simple growth property implicitly; the full details are contained in [20,  Section 6]. Note that there, the only property of the joint spectral radius used is [20,  Equation (7.1)].
2. The given expressions for the Fourier coefficients allow their computation with high precision; see [20,  Part IV]. Furthermore, an implementation is available at https://gitlab.com/dakrenn/regular-sequence-fluctuations. We will use this implementation to compute the Fourier coefficients for the examples in Sects. 5, 6 and 7.
3. The motivation for analyzing the summatory function instead of the sequence itself is the following: The asymptotic behavior of regular sequences is often not smooth (which would imply that in any asymptotic expansion as given in [20], everything is absorbed by the error term), whereas the asymptotic behavior of the summatory function is. However, it is also possible to apply Theorem F to a *q*-regular sequence *x* itself: Let us write  So *x* can be represented as the summatory function of the sequence of differences
  $$\begin{aligned} f(n) := x(n+1) - x(n), \end{aligned}$$
  which is again *q*-regular by [1,  Theorems 2.5 and 2.6]. Consequently, applying Theorem F to *f* yields an asymptotic analysis for  which differs from the asymptotic behavior of *x* only by an additive constant. $\square$

### Spectral Results in the General Case

In this section, we show that in most cases, the asymptotic behavior of a regular sequence can be deduced directly from a linear representation which is valid from some offset $n_0\ge 1 > 0$. In these cases, it is not necessary to use Theorem B to construct an augmented linear representation valid for all non-negative integers. So, we will assume that $n_0\ge 1$ because otherwise, there is nothing to do.

We first consider the significant eigenvalues and then the significant joint spectral radii (significant with respect to Theorem F).

#### Proposition 4.4

Let $A_{0}$, ..., $A_{q-1}$, ${\widetilde{A}}_{0}$, ..., ${\widetilde{A}}_{q-1}$ and $n_{0}$ as in Theorem B. Assume that $n_0\ge 1$. SetThen  holds. In particular,

- if $n_{0} = 1$, then  and for all , we have $m_C(\lambda ) = m_{{\widetilde{C}}}(\lambda )$; and
- if $n_{0} \ge 2$, then  and for all , we have $m_C(\lambda ) = m_{{\widetilde{C}}}(\lambda )$.

Before stating the second result, we state a lemma dealing with the simple growth property for sets of block triangular matrices. This is a refinement of Jungers [25,  Proposition 1.5], which deals with the joint spectral radius only (here restated as the first statement of the lemma).

#### Lemma 4.5

Let ${\mathcal {G}}$ be a finite set of $(D_1+D_2+\cdots +D_s)\times (D_1+D_2+\cdots + D_s)$ block upper triangular matrices. For $G\in {\mathcal {G}}$ write

$$\begin{aligned} G = \begin{pmatrix} G^{(11)}&{}\quad G^{(12)}&{}\quad \ldots &{}\quad G^{(1s)}\\ 0&{}\quad G^{(22)}&{}\quad \ldots &{}\quad G^{(2s)}\\ \vdots &{}\quad \vdots &{}\quad \ddots &{}\quad \vdots \\ 0&{}\quad 0&{}\quad \ldots &{}\quad G^{(ss)} \end{pmatrix} \end{aligned}$$

where the block $G^{(ij)}$ is a $D_i\times D_j$ matrix for $1\!\le \! i\!\le \! j\!\le \! s$. Set  ${\mathcal {G}}^{(i)}\!:=\! \{G^{(ii)} \mid G\!\in \!{\mathcal {G}}\}$. Then $\rho ({\mathcal {G}})=\max _{1\le i\le s}\rho ({\mathcal {G}}^{(i)})$.

If there is a unique $i_0\in \{1, \ldots , s\}$ such that $\rho ({\mathcal {G}}^{(i_0)})=\rho ({\mathcal {G}})$ and ${\mathcal {G}}^{(i_0)}$ has the simple growth property, then ${\mathcal {G}}$ has the simple growth property.

We now state the result on the joint spectral radius in the context of Theorem B.

#### Proposition 4.6

Let ,  and  be the sets of matrices and $n_0$ the offset as given in Theorem B, and assume $n_0\ge 1$. Then the joint spectral radii of $\widetilde{{\mathcal {A}}}$ and ${\mathcal {J}}$ satisfy4.5respectively. In particular, if $\rho ({\mathcal {A}}) \ge 1$ holds, then we have $\rho (\widetilde{{\mathcal {A}}}) = \rho ({\mathcal {A}})$.

Furthermore, if $\rho ({\mathcal {A}}) > 1$ holds and ${\mathcal {A}}$ has the simple growth property, then $\widetilde{{\mathcal {A}}}$ has the simple growth property.

Combining Propositions 4.4 and 4.6 with Theorem F implies that the asymptotics can also be determined by using the matrices $A_{0}$, ..., $A_{q-1}$ (which do not contain the correction for the offset; see Theorem B) instead of the matrices ${\widetilde{A}}_{0}$, ..., ${\widetilde{A}}_{q-1}$ from the linear representation.

Note that if $\rho ({\mathcal {A}})<1$, then the error in (4.1) is *o*(1). This implies that adding constants (created by correction terms if the recurrence relation is not valid for some $n\ge 0$) is visible in the asymptotic expansion.

### Spectral Results in the Special Case

Next, we are interested in the eigenvalues of the matrix $C = \sum _{0\le r < q}A_{r}$ for the special case. It turns out that the eigenvalues of *C* can be obtained from the recurrence relations (3.1) more directly than via the detour to linear representations.

Note that also here we assume the offset to be $n_{0} = 0$ for the sake of readability, analogous to Sect. 3.3. The following results can be generalized easily for arbitrary offset.

#### Proposition 4.7

Let $A_{0}$, ..., $A_{q-1}$ and $B_{0}$, ..., $B_{q-1}$ be the matrices as given in Theorem E, let $M = m+1$ and *m* be the exponents of the corresponding *q*-recursive sequence with $m \ge 1$ and set $C = \sum _{0\le r < q}A_{r}$. Then we haveMoreover, we have $m_{C}(\lambda ) = m_{B_{0} + \cdots + B_{q-1}}(\lambda )$ for all .

#### Proposition 4.8

Let ,  and  the sets of matrices as given in Theorem E. Then the joint spectral radii of ${\mathcal {A}}$ and ${\mathcal {J}}$ satisfy

$$\begin{aligned} \rho ({\mathcal {A}}) = \rho ({\mathcal {B}}) \text { and } \rho ({\mathcal {J}}) = 0, \end{aligned}$$

respectively.

Furthermore, if $\rho ({\mathcal {B}}) > 0$ holds and ${\mathcal {B}}$ has the simple growth property, then ${\mathcal {A}}$ has the simple growth property.

The two propositions of this section provide the possibility to obtain the asymptotics of the summatory function without knowing a linear representation of the sequence; the asymptotics are fully determined by the matrices $B_{0}$, ..., $B_{q-1}$.

### Functional Equation for the Dirichlet Series in the Special Case

Theorem F indicates that functional equations for the Dirichlet series corresponding to the sequence of interest are essential for computing Fourier coefficients of the periodic fluctuations. We now give an variant for our special case of a *q*-recursive sequence which does not require constructing the *q*-linear representation first.

#### Proposition 4.9

Let *x* be a *q*-recursive sequence with exponents $M=m+1$ and *m*, index shift bounds $\ell = 0$ and $u = q^{m} - 1$ and coefficients $(c_{j,k})_{0\le j< q^{m+1}, 0\le k < q^{m}}$, and let $B_{0}$, ..., $B_{q-1}$ be the matrices introduced in (3.11). Let $\rho > 0$ be such that $x(n) = O(n^{\log _{q}R})$ as $n \rightarrow \infty$ holds for all $R > \rho$, and let $\eta \ge 1$ be an integer. We define the Dirichlet series4.6for $0\le j < q^{m}$ and $\Re s>\log _q \rho + 1$ and set

$$\begin{aligned} {\mathcal {X}}(s) {{:=}} \begin{pmatrix} {\mathcal {X}}_{0}(s)\\ \vdots \\ {\mathcal {X}}_{q^{m}-1}(s) \end{pmatrix}. \end{aligned}$$

Then the functional equation

4.7 $$\begin{aligned} \bigl (I - q^{-s}(B_{0} + \cdots + B_{q-1})\bigr ){\mathcal {X}}(s) = \begin{pmatrix} {\mathcal {Y}}_{0}(s)\\ \vdots \\ {\mathcal {Y}}_{q^{m}-1}(s) \end{pmatrix} \end{aligned}$$

holds for $\Re s > \log _{q}\rho$ with4.8Moreover, ${\mathcal {Y}}_{j}(s)$ is analytic for $\Re s>\log _q \rho$ and all $0\le j < q^{m}$, and, in particular, ${\mathcal {X}}(s)$ is meromorphic for $\Re s>\log _q \rho$ and can only have poles *s* where $q^{s}\in \sigma (B_{0} + \cdots + B_{q-1})$.

## Stern’s Diatomic Sequence

### Introduction of the Sequence

We start our detailed study of particular sequences by studying a sequence which has a long history, namely the so-called^10^*Stern’s diatomic sequence*; see [36,  A002487]. After its earliest introduction by Stern [35] in 1858, the sequence has been studied thoroughly; see Northshield [33] and the references therein, and also Coons and Shallit [10], Leroy, Rigo and Stipulanti [28].

Stern’s diatomic sequence *d* is defined by^11^$d(0) = 0$, $d(1) = 1$ and

5.1a $$\begin{aligned} d(2n)&= d(n), \end{aligned}$$

5.1b $$\begin{aligned} d(2n + 1)&= d(n) + d(n+1) \end{aligned}$$

for all $n \ge 0$. The first few terms of *d* are given in Table 2.

**Table 2:** First few elements of Stern’s diatomic sequence *d*

*n*	0	1	2	3	4	5	6	7	8	9	10	11	12	13	14	15
*d*(*n*)	0	1	1	2	1	3	2	3	1	4	3	5	2	5	3	4

### Combinatorial Interpretations of the Sequence

There are several combinatorial interpretations of Stern’s diatomic sequence. In the following, we give a short overview of the most interesting connections to combinatorial settings.

1. Let us call the word $d_{L-1}\ldots d_{0}$ over the alphabet  a *hyperbinary representation* of some $n\in {\mathbb {N}}_{0}$ if $n = \sum _{0\le i<L}d_{i}2^{i}$ and $d_{L-1}\ne 0$. Then the number of different hyperbinary representations of *n* is equal to $d(n+1)$ for all $n \ge 0$; see Northshield [33,  Theorem 3.1].
2. Let $\genfrac\rbrace \lbrace {0.0pt}{}{n}{r}$ denote the *Stirling partition numbers*, i.e., $\genfrac\rbrace \lbrace {0.0pt}{}{n}{r}$ is the number of different partitions of the set  in exactly *r* non-empty subsets. Then *d*(*n*) equals the number of integers $r\in {\mathbb {N}}_{0}$ such that $\genfrac\rbrace \lbrace {0.0pt}{}{n}{2r}$ is even and non-zero; see Carlitz [8].
3. Let *F*(*n*) be the *n*th *Fibonacci number*. Then *d*(*n*) is equal to the number of different representations of *n* as a sum of distinct Fibonacci numbers *F*(2*k*) with $k\in {\mathbb {N}}_{0}$; see Bicknell-Johnson [6].
4. An *alternating bit set* of some integer $n\in {\mathbb {N}}_{0}$ is a subset *A* of the positions in the binary expansion of *n* such that
  the bits of the binary expansion of *n* at positions which lie in *A* are alternating between 1 and 0,
  the most significant bit at a position which lies in *A* is a 1, and
  the least significant bit at a position which lies in *A* is a 0.
  In particular, we also allow $A = \emptyset$ to be an alternating bit set. Note that this definition implies that every alternating bit set has even cardinality. Then the number of different alternating bit sets of *n* is equal to $d(n+1)$; see Finch [14,  Section 2.16.3].
5. There is a relation to the well-known *Towers of Hanoi*; see Hinz, Klavžar, Milutinović, Parisse and Petr [23].

Thus, the asymptotic analysis of the summatory function of Stern’s diatomic sequence is indeed well-motivated, also from a combinatorial point of view.

### Regularity and a Linear Representation

In order to be able to apply Theorem F for the asymptotic analysis of the summatory function, the sequence *d* has to be recursive. Due to the definition of the sequence in (5.1), it is clear that *d* is 2-recursive with exponents $M=1$ and $m=0$, index shift bounds $\ell = 0$ and $u = 1$, and offset $n_0=0$. Thus, it is also 2-regular by Theorem A. Note that Theorem E is not applicable: The term $d(n+1)$ appears in (5.1b) and therefore, the upper index shift bound *u* needs to be at least 1, whereas Theorem E only allows 0 as an upper index shift bound in the case $m=0$. So we use Theorem A to obtain a 2-linear representation $(A_{0}, A_{1}, v)$ of *d*: The vector-valued sequence *v* is given by

$$\begin{aligned} v = \begin{pmatrix} d\\ d\circ (n\mapsto n+1)\\ d\circ (n\mapsto n+2) \end{pmatrix}, \end{aligned}$$

and the matrices are given by

$$\begin{aligned} A_{0} = \begin{pmatrix} 1 &{}\quad 0 &{}\quad 0\\ 1 &{}\quad 1 &{}\quad 0\\ 0 &{}\quad 1 &{}\quad 0 \end{pmatrix} \text { and } A_{1} = \begin{pmatrix} 1 &{}\quad 1 &{}\quad 0\\ 0 &{}\quad 1 &{}\quad 0\\ 0 &{}\quad 1 &{}\quad 1 \end{pmatrix}. \end{aligned}$$

The correctness of the recurrence relations $v(2n) = A_{0}v(n)$ and $v(2n + 1) = A_{1}v(n)$ for all $n \ge 0$ can easily be verified by using (5.1).

As in Example 3.7, we can see that $d\circ (n\mapsto n+2)$ is actually not needed in the linear representation, which is due to the fact that the coefficient of $d(n + 1)$ in the recurrence relation (5.1a) is zero. This implies that $(A_{0}',A_{1}',v')$ with

$$\begin{aligned} v' = \begin{pmatrix} d\\ d\circ (n\mapsto n+1) \end{pmatrix} \end{aligned}$$

as well as

$$\begin{aligned} A_{0}' = \begin{pmatrix} 1 &{}\quad 0\\ 1 &{}\quad 1 \end{pmatrix} \text { and } A_{1}' = \begin{pmatrix} 1 &{}\quad 1\\ 0 &{}\quad 1 \end{pmatrix} \end{aligned}$$

is also a 2-linear representation of *d*.

By applying the minimization algorithm mentioned in Remark 3.6, we see that this is the smallest possible 2-linear representation of *d*.

### Asymptotics

Let ${\mathcal {V}}(s)$ denote the Dirichlet series corresponding to $v'$, i.e.,

$$\begin{aligned} {\mathcal {V}}(s) = \sum _{n\ge 1}n^{-s}v'(n), \end{aligned}$$

and let $C = A_{0}' + A_{1}'$. In the following theorem, we state the main result of this section: We give an asymptotic formula for the summatory function of *d* as well as a functional equation for ${\mathcal {V}}(s)$.

#### Theorem G

(Asymptotics for Stern’s diatomic sequence) The summatory function *D* of Stern’s diatomic sequence *d* satisfies5.2as $N \rightarrow \infty$, where $\kappa = \log _{2}3 = 1.5849625007211\ldots$, $\varphi = \frac{1+\sqrt{5}}{2} = 1.6180339887498\ldots$ is the golden ratio, $\log _{2}\varphi = 0.69424191363061\ldots$ and $\Phi _{D}$ is a 1-periodic continuous function which is Hölder continuous with any exponent smaller than $\kappa -\log _{2}\varphi$. The Fourier coefficients of $\Phi _{D}$ can be computed efficiently.

Furthermore, the Dirichlet series ${\mathcal {V}}(s)$ satisfies the functional equation

5.3 $$\begin{aligned} (I - 2^{-s}C){\mathcal {V}}(s) = v'(1) + 2^{-s}A_{1}'\sum _{k\ge 1}\frac{1}{2^{k}}\left( {\begin{array}{c}-s\\ k\end{array}}\right) {\mathcal {V}}(s+k) \end{aligned}$$

for all $\Re s > \log _{2}\varphi$. Both sides of Eq. (5.3) are analytic for $\Re s > \log _{2}\varphi$, and, in particular, ${\mathcal {V}}(s)$ is meromorphic for $\Re s>\log _2\varphi$ and can only have at most simple poles $s = \log _{2}3 + \frac{2i\pi \mu }{\log 2}$ with $\mu \in {\mathbb {Z}}$.

**Fig. 1:**
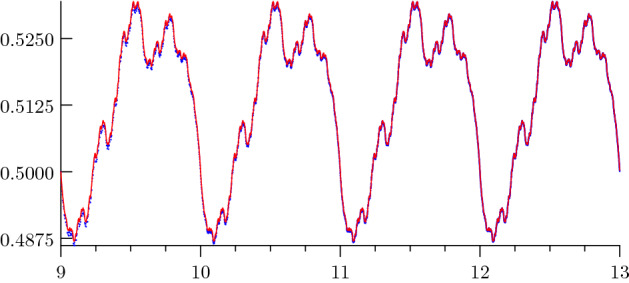
Fluctuation in the main term of the asymptotic expansion of the summatory function *D*. The plot shows the periodic fluctuation $\Phi _{D}(u)$ approximated by its Fourier series of degree 2000 (red) as well as the function $D(2^{u})/2^{\kappa u}$ (blue)

Table 3 shows the first few Fourier coefficients and Fig. 1 a plot of the periodic fluctuation of Theorem G.

**Table 3:** First few Fourier Coefficients of $\Phi _{D}$

$\mu$	$\varphi _{\mu }$
0	0.5129922721107177789989881697483
1	$-0.00572340619479957230984532582323 + 0.00692635056470554871320794780023i$
2	$0.00024322678681282580951796870908 + 0.00296266191012688412725699259509i$
3	$-0.00145239145783579607592238228126 + 0.00117965322085442917547658711471i$
4	$0.00111195666191700704424207971541 + 0.00018518355971470343780812186861i$
5	$-0.00046732929957426516792963051204 + 0.00050425058689999021735711128987i$
6	$-0.00044953390461558932213468137492 + 0.00048773649732968174101103217106i$
7	$0.00036329328164895877338262637843 + 0.00035534416834062145852032394307i$
8	$-0.00016679033186561839463311958967 - 0.00043694014091729453542478927729i$
9	$0.00030367683575578278808761185183 + 0.00009371153567156005005069054904i$
10	$-0.00009911479960205956796299031716 + 0.00019462735102739460438023334462i$

#### Proof of Theorem G

We use Theorem F with the linear representation $(A_{0}', A_{1}', v')$ and work out the parameters needed in the theorem. Recall that $C = A_{0}' + A_{1}'$.

*Joint Spectral Radius.* We determine the joint spectral radius of $A_{0}'$ and $A_{1}'$. As one matrix is the transpose of the other, the spectral norm of each of them equals the square root of the dominant eigenvalue of their product. The maximal spectral norm of the matrices is an upper bound for the joint spectral radius; the square root of the dominant eigenvalue of their product is a lower bound for the joint spectral radius. As both bounds are equal, the joint spectral radius equals the spectral norm. It turns out that this spectral norm equals $\varphi = \frac{1 + \sqrt{5}}{2}$.

*Finiteness Property.* The finiteness property for $A_{0}'$ and $A_{1}'$ is satisfied with respect to the spectral norm, which can be seen by considering exactly one factor $A_{0}'$ or $A_{1}'$. Thus, we choose $R = \varphi$.

*Eigenvalues.* The spectrum of *C* is given by . Furthermore, it is clear that all eigenvalues are simple and thus, $m_{C}(3) = 1$.

Applying Theorem F yields the result. $\square$

It will turn out during the proof of Theorem K that a slight modification of the summatory function leads to an exact formula:

#### Corollary H

With the notations of Theorem G, we have

## Number of Non-Zero Entries in a Generalized Pascal’s Triangle

### Introduction of the Sequence

The first two authors of this article have studied Pascal’s rhombus as one possible generalization of Pascal’s triangle in [20] as well as in [22] together with Prodinger. In particular, they analyzed the asymptotic behavior of the number of odd entries in the *n*th row of Pascal’s rhombus.

Here, we consider a generalization of Pascal’s triangle to binomial coefficients of words. This generalization was first introduced by Leroy, Rigo and Stipulanti in [27]. We in particular study the sequence counting the number of non-zero elements in each row (see [36] except for the initial value), which was investigated in detail by the same authors in [29] and [30], and provide an asymptotic result for the summatory function. Our result coincides with the result in [30]. In contrast to [30], we additionally provide the periodic fluctuation that occurs in the asymptotics by determining its Fourier coefficients. This completes the full picture of the summatory function.

We start with the following definition; also see Lothaire [31,  Chapter 6] for more details on binomial coefficients of words.

#### Definition 6.1

(*Scattered Subword, Binomial Coefficients of Words*) Let $u = u_{1}\ldots u_{j}$ and $v = v_{1}\dots v_{k}$ be two words over the same alphabet.

- We say that *v* is a *scattered subword* of *u* if there exists a strictly increasing mapping  with $u_{\pi (i)} = v_{i}$ for all $1\le i\le k$. We call $\pi$ an *occurrence* of *v* as a scattered subword of *u*.
- The *binomial coefficient* of *u* and *v*, denoted by $$\left( {\begin{array}{c}u\\ v\end{array}}\right) $$, is defined as the number of different occurrences of *v* as a scattered subword of *u*.

For example, we consider the words $u = u_{1}u_{2}u_{3}u_{4}u_{5}u_{6} = 110010$ and $v = v_{1}v_{2} = 10$ over the alphabet . Then we have

6.1 $$\begin{aligned} \left( {\begin{array}{c}110010\\ 10\end{array}}\right) = 7 \end{aligned}$$

because there are exactly seven possibilities to represent *v* as a scattered subword of *u*, namely

$$\begin{aligned} u_{1}u_{3} = u_{1}u_{4} = u_{1}u_{6} = u_{2}u_{3} = u_{2}u_{4} = u_{2}u_{6} = u_{5}u_{6} = v. \end{aligned}$$

Note that the classical binomial coefficient for two integers *n*, $k\in {\mathbb {N}}_{0}$ can be obtained by the identity

$$\begin{aligned} \left( {\begin{array}{c}1^{n}\\ 1^{k}\end{array}}\right) = \left( {\begin{array}{c}n\\ k\end{array}}\right) , \end{aligned}$$

where $1^{n}$ denotes the word consisting of *n* ones.

Next, we define the *generalized Pascal’s triangle* ${\mathcal {P}}_{2}$ as an infinite matrix as follows: The entry in the *n*th row and *k*th column of ${\mathcal {P}}_{2}$ is given by $$\left( {\begin{array}{c}(n)_{2}\\ (k)_{2}\end{array}}\right) $$, where $(n)_{2}$ denotes the binary expansion of some $n\in {\mathbb {N}}_{0}$, i.e.,

$$\begin{aligned} {\mathcal {P}}_{2} {{:=}} \Biggl (\left( {\begin{array}{c}(n)_{2}\\ (k)_{2}\end{array}}\right) \Biggr )_{\begin{array}{c} n\ge 0\\ k\ge 0 \end{array}}. \end{aligned}$$

**Fig. 2:**
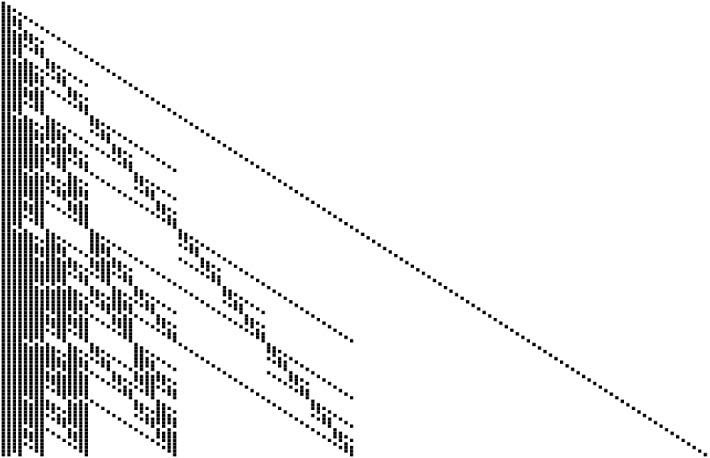
Non-zero elements in the generalized Pascal’s triangle ${\mathcal {P}}_2$

Observe that $$\left( {\begin{array}{c}(n)_{2}\\ (0)_{2}\end{array}}\right) = 1$$ and $$\left( {\begin{array}{c}(n)_{2}\\ (n)_{2}\end{array}}\right) = 1$$ hold for all $n \ge 0$. We let *z* denote the sequence of interest and define *z*(*n*) as the number of non-zero elements in the *n*th row of ${\mathcal {P}}_{2}$. The first few values of ${\mathcal {P}}_{2}$ are given in Table 4, and the last column shows the first few values of *z*. Figure 2 illustrates the non-zero elements in ${\mathcal {P}}_{2}$.

**Table 4:**
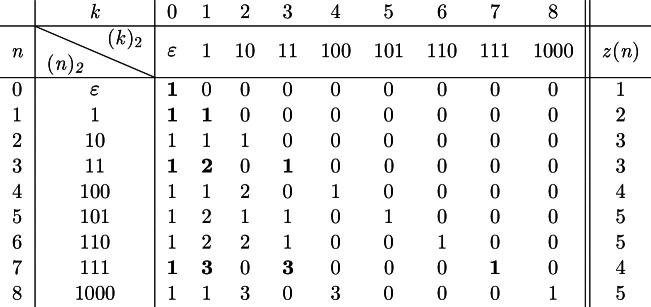
The first few elements of the generalized Pascal’s triangle ${\mathcal {P}}_2$ as well as the corresponding number of non-zero elements in each row. The values of the ordinary Pascal’s triangle are printed in bold

The following result by Leroy, Rigo and Stipulanti [29] provides a (at least on the first glance) surprising connection between the number of non-zero elements in ${\mathcal {P}}_{2}$ and Stern’s diatomic sequence.

#### Theorem I

(Leroy–Rigo–Stipulanti  [ Section 4] LeroyspsRigospsStipulanti:2017:nonspszerospsgeneralizedspspascalspstriangle) The sequence *z* satisfies the relation

$$\begin{aligned} z(n) = d(2n + 1) \end{aligned}$$

for all $n \ge 0$, where *d* is Stern’s diatomic sequence as studied in Sect. 5.

### Regularity and a Linear Representation

In principle, Theorem I and the results on the asymptotics of Stern’s diatomic sequence given in Sect. 5 could be used to determine the asymptotics of *z*. However, it turns out that the error term in the asymptotic expansion of *z* vanishes. In order to show this, the results of Sect. 5 are not sufficient, and we need to have a closer look at the linear representation of *z*. Theorem I does not suffice for this purpose, so instead we intend to present three different possibilities for obtaining a linear representation. The first one will give some more details on the reduction via Theorem I, while the others will be based on the following result, also by Leroy, Rigo and Stipulanti.

#### Theorem J

(Leroy–Rigo–Stipulanti  [29,  Theorem 21]) The sequence *z* satisfies the recurrence relations

6.2a $$\begin{aligned} z(2n + 1)&= 3z(n) - z(2n), \end{aligned}$$

6.2b $$\begin{aligned} z(4n)&= - z(n) + 2z(2n), \end{aligned}$$

6.2c $$\begin{aligned} z(4n + 2)&= 4z(n) - z(2n) \end{aligned}$$

for all $$n \ge 0$$.

As already mentioned, the previous theorem as well as Theorem I provide several possibilities to find a linear representation of *z*, and we discuss three of them. As a side product of the second approach, it will also be clear why *z* is a recursive sequence and therefore fits into our framework.

**Approach 1.**

First of all, it is worth mentioning that we can use Theorem I to obtain a 2-linear representation: Since Stern’s diatomic sequence *d* is 2-regular and $z(n) = d(2n + 1)$ holds for all $n\in {\mathbb {N}}_{0}$, the 2-regularity of *z* follows by Allouche and Shallit [1,  Theorem 2.6]. In the proof of [1,  Theorem 2.6] we also find a description for the construction of a 2-linear representation of *z* by using the linear representation of *d*. We do not want to go into detail here.

**Approach 2.**

The recurrence relations in Theorem J are not directly in line with the desired relations in the framework of *q*-recursive sequences as given in (3.1). This second approach does not only lead to a desired linear representation of *z*, but it also illustrates how recurrence relations as given in (6.2) can be disentangled in order to obtain appropriate recurrence relations for a *q*-recursive sequence. In fact, we will show that the sequence *z* is 2-recursive, which directly implies that it is also 2-regular due to Theorem E.

For this purpose, consider the system of equations

6.3 $$\begin{aligned} \begin{pmatrix} -3 &{} 1 &{} 1 &{} 0 &{} 0 &{} 0 &{} 0 \\ 1 &{} -2 &{} 0 &{} 1 &{} 0 &{} 0 &{} 0 \\ -4 &{} 1 &{} 0 &{} 0 &{} 0 &{} 1 &{} 0 \\ 0 &{} -3 &{} 0 &{} 1 &{} 1 &{} 0 &{} 0 \\ 0 &{} 0 &{} -3 &{} 0 &{} 0 &{} 1 &{} 1 \end{pmatrix} \begin{pmatrix} z\\ z\circ (n\mapsto 2n)\\ z\circ (n\mapsto 2n + 1)\\ z\circ (n\mapsto 4n)\\ z\circ (n\mapsto 4n + 1)\\ z\circ (n\mapsto 4n + 2)\\ z\circ (n\mapsto 4n + 3)\\ \end{pmatrix} = 0, \end{aligned}$$

where the first three rows correspond to the relations given in Theorem J and the last two rows arise from (6.2a) by replacing *n* by 2*n* and by $2n+1$, respectively.

We want to get a representation of *z* as a 2-recursive sequence. It turns out that we can achieve such a sequence with exponents $M = 2$ and $m = 1$, so we need explicit expressions for $z\circ (n\mapsto 4n)$, $z\circ (n\mapsto 4n+1)$, $z\circ (n\mapsto 4n+2)$ and $z\circ (n\mapsto 4n+3)$ (corresponding to the last four columns of the matrix). We also want these expressions to be free from *z* itself (corresponding to the first column of the matrix), so we transform the system in such a way that an identity matrix appears in these columns. Indeed, we multiply the system from the left with the inverse of the matrix formed by these five columns and obtain6.4Here the first four rows give the system

$$\begin{aligned} z(4n)&= \frac{5}{3}z(2n) - \frac{1}{3}z(2n + 1), \\ z(4n + 1)&= \frac{4}{3}z(2n) + \frac{1}{3}z(2n + 1),\\ z(4n + 2)&= \frac{1}{3}z(2n) + \frac{4}{3}z(2n + 1),\\ z(4n + 3)&= -\frac{1}{3}z(2n) + \frac{5}{3}z(2n + 1) \end{aligned}$$

for $n \ge 0$, which is a representation of *z* as a 2-recursive sequence with offset $n_{0} = 0$, exponents $M = 2$ and $m = 1$ and index shift bounds $\ell =0$ and $u=1$. The last row of (6.4) can be omitted.

The matrices $B_{0}$ and $B_{1}$ as introduced in (3.11) are given bysee also the marked submatrices in (6.4). We can now apply Theorem E and obtain a 2-linear representation $(A_{0}, A_{1}, v)$ of *z* with dimension 3: The vector-valued sequence *v* is given by

$$\begin{aligned} v = \begin{pmatrix} z\\ z\circ (n\mapsto 2n)\\ z\circ (n\mapsto 2n + 1) \end{pmatrix}, \end{aligned}$$

and due to (3.14), the matrices $A_{0}$ and $A_{1}$ have the formNote that by (6.2a) we know that the three sequences contained in *v* are linearly dependent. This means that we could replace $z\circ (n\mapsto 2n + 1)$ by a linear combination of the other two sequences and obtain a linear representation of dimension 2. However, we prefer to derive a linear representation with this vector directly as Approach 3 below.

**Approach 3.**

Finally, we can also derive a linear representation of *z* directly from the recurrence relations given in Theorem J. By setting

6.5 $$\begin{aligned} v = \begin{pmatrix} z\\ z\circ (n\mapsto 2n) \end{pmatrix}, \end{aligned}$$

we obtain 6.6aas well as6.6b for all $n\in {\mathbb {N}}_{0}$. See also [29, Corollary 22].

By applying the minimization algorithm mentioned in Remark 3.6, we see that this is the smallest possible 2-linear representation of *z*.

### Full Asymptotics

We now come to the main theorem of this section: We provide an explicit formula for the summatory function $Z(N) = \sum _{0\le n < N}z(n)$.

#### Theorem K

(Full asymptotics for the number of non-zero elements in the generalized Pascal’s triangle ${\mathcal {P}}_{2}$) The summatory function *Z* of the sequence *z* equals6.7for $N\ge 1$ with $\kappa = \log _{2}3$ and $\Phi _{Z} = 2\Phi _{D}$, where $\Phi _{D}$ is the periodic fluctuation which occurs in the asymptotic expansion of Stern’s diatomic sequence in Theorem G.

**Fig. 3:**
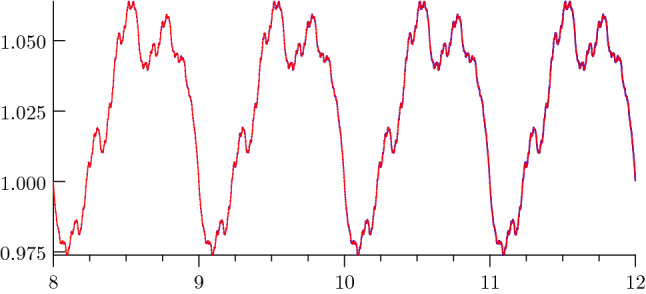
Fluctuation in the asymptotic expansion of the summatory function *Z*. The plot shows the periodic fluctuation $\Phi _{Z}(u) = 2\Phi _{D}(u)$ approximated by its Fourier polynomial of degree 2000 (red) as well as the function $Z(2^{u})/2^{\kappa u}$ (blue)

For a plot of $\Phi _{Z}$ see Fig. 3.

#### Proof of Theorem K

We use the 2-dimensional linear representation $(A_{0}, A_{1}, v)$ given in (6.5) and (6.6), and we want to apply [20,  Theorem B] instead of Theorem F in order to get rid of the error term coming from Theorem F.

The left eigenvectors of the matrix

$$\begin{aligned} C = A_{0} + A_{1} = \begin{pmatrix} 3 &{} 0\\ 3 &{} 1 \end{pmatrix} \end{aligned}$$

are given by $v_{1} = (1, -2/3)$, which corresponds to the eigenvalue $\lambda = 1$, and $v_{3} = (1, 0)$, which corresponds to the eigenvalue $\lambda = 3$. Since the vector $v_{3}$ is equal to the left “choice vector” $e_{1}$ of the linear representation (see (2.1)), it follows from [20,  Section 6.3] that the eigenvalue $\lambda = 3$ is the only one which contributes to the summatory function.

By the above observations, the matrix *C* has the Jordan decomposition $C = T^{-1}JT$ with

$$\begin{aligned} J = \begin{pmatrix} 3 &{}\quad 0\\ 0 &{}\quad 1 \end{pmatrix} \text { and}\; T = \begin{pmatrix} 1 &{}\quad 0\\ 1 &{}\quad -2/3 \end{pmatrix}. \end{aligned}$$

With the notation introduced in [20,  Section 6.2], we moreover have

$$\begin{aligned} D = \begin{pmatrix} 1 &{}\quad 0\\ 0 &{}\quad 0 \end{pmatrix} \text { and}\; C' = T^{-1}DJT = \begin{pmatrix} 3 &{}\quad 0\\ 9/2 &{}\quad 0 \end{pmatrix} \end{aligned}$$

as well as

$$\begin{aligned} K = T^{-1}DT(I_{2} - C')^{-1}(I_{2} - A_{0}) = \begin{pmatrix} -1/2 &{} 1/2\\ -3/4 &{} 3/4 \end{pmatrix}, \end{aligned}$$

where $I_{2}$ denotes the $2\times 2$ identity matrix. Due to Theorem I and the second paragraph of the proof of Theorem G, we can choose $R = \varphi = \frac{1 + \sqrt{5}}{2}$. Now we are ready to apply [20,  Theorem B] for the eigenvalue $\lambda = 3$. By noting $Kv(0) = 0$, we obtain

6.8 $$\begin{aligned} Z(N) = N^{\kappa }\cdot \Phi _{Z}(\{ \log _{2}N \}), \end{aligned}$$

where $\Phi _{Z}$ is a 1-periodic function which is Hölder continuous with any exponent smaller than $\kappa -\log _{2}\varphi$.

Finally, we argue that $\Phi _{Z} = 2\Phi _{D}$. Due to Theorem I and (5.1b), we have6.9as $N \rightarrow \infty$. Applying Theorem G on *D*(*N*) and combining the result with (6.8) yields

$$\begin{aligned} N^{\kappa }\cdot \Phi _{Z}(\{ \log _{2}N \}) = N^{\kappa }\cdot 2\Phi _{D}(\{ \log _{2}N \}) + O(N^{\log _{2}\varphi }). \end{aligned}$$

Since the set  is dense in the interval [0, 1] and both $\Phi _{Z}$ and $2\Phi _{D}$ are continuous, they have to be equal. This concludes the proof. $\square$

We are now able to prove Corollary H.

#### Proof of Corollary H

The statement of the corollary follows from combining (6.9) with Theorem K. $\square$

## Number of Unbordered Factors in the Thue–Morse Sequence

### Introduction of the Sequence

The Thue–Morse sequence *t* is the parity sequence of the binary sum of digits; see [36,  A010060]. In this section, we study the number of unbordered factors in the Thue–Morse sequence, which are a special kind of subsequences of *t*. Unbordered factors of the Thue–Morse sequence were first investigated by Currie and Saari in [12]. Some more interesting insights concerning this special type of factors can, for example, be found in Charlier, Rampersad and Shallit [9] and Goč, Henshall and Shallit [16]; see also Goč, Rampersad, Rigo and Salimov [18].

Before we formally define the sequence of study, we give the following definitions from combinatorics on words, which can, for example, also be found in Lothaire [32].

#### Definition 7.1

(Unbordered Factor) Let ${\mathcal {A}}$ be an alphabet and $x:{\mathbb {N}}_{0}\rightarrow {\mathcal {A}}$ a sequence.

1. For integers $0\le i \le j$, we let $x[i\,.\,.\,j]$ denote the subword $x(i)x(i+1)\ldots x(j-1)x(j)$ of the infinite word $x(0)x(1)x(2)x(3)\dots$.
2. We say that a word $w\in {\mathcal {A}}^{\star }$ is a *factor of* *x* if there exist integers $0\le i\le j$ such that $w = x[i\,.\,.\,j]$.
3. A word $w\in {\mathcal {A}}^{\star }$ is said to be *bordered* if there exists a non-empty word $v\ne w$ which is both a prefix and a suffix of *w*. In this case we call *v* a *border* of *w*. If *w* is not bordered, then it is said to be *unbordered*.

In particular, this implies that the empty word $\varepsilon$ as well as every word of length 1 is unbordered. Moreover, a word $w = ab$ of length 2 with *a*, $b\in {\mathcal {A}}$ is unbordered if and only if $a\ne b$.

We illustrate the previous definitions by the following example.

#### Example 7.2

(Some (Un)Bordered Factors of the Thue–Morse Sequence) If we write the Thue–Morse sequence as an infinite word $t = t(0)t(1)t(2)\dots$, then it starts with

$$\begin{aligned} t = 01101001\,10010110\,10010110\,01101001\dots . \end{aligned}$$

We visually structured the sequence into blocks of eight letters in order to emphasize some of its properties. Some bordered factors of *t* are given in Table 5, whereas the factors in Table 6 are unbordered.

It is easy to check that Table 6 contains a complete list of the unbordered factors of the Thue–Morse sequence with length smaller than 4.

**Table 5:** Some bordered factors of the Thue–Morse sequence *t*

bordered factor	border	length
$t[5\,.\,.\,6] = 00$	0	2
$t[1\,.\,.\,2] = 11$	1	2
$t[3\,.\,.\,5] = 010$	0	3
$t[2\,.\,.\,4] = 101$	1	3
$t[2\,.\,.\,5] = 1010$	10	4
$t[0\,.\,.\,9] = 0110100110$	0110	10

**Table 6:** All unbordered factors of the Thue–Morse sequence *t* up to length 3

unbordered factor	length
$\varepsilon$	0
$t[0\,.\,.\,0] = 0$	1
$t[1\,.\,.\,1] = 1$	1
$t[0\,.\,.\,1] = 01$	2
$t[2\,.\,.\,3] = 10$	2
$t[0\,.\,.\,2] = 011$	3
$t[1\,.\,.\,3] = 110$	3
$t[4\,.\,.\,6] = 100$	3
$t[5\,.\,.\,7] = 001$	3

Now the following question may arise: Is there an unbordered factor of *t* with length *n* for every $n \ge 0$? Currie and Saarie showed in [12] that *t* has an unbordered factor of length *n* if . However, since

$$\begin{aligned} t[39\,.\,.\,69] = 0011010010110100110010110100101 \end{aligned}$$

is an unbordered factor of length 31 in *t*, the given condition is not necessary. Goč, Henshall and Shallit finally proved the following characterization.

#### Theorem L

(Goč, Henshall and Shallit  [16,  Theorem 4]) There is an unbordered factor of length *n* in *t* if and only if $(n)_{2}\notin 1(01^{*}0)^{*}10^{*}1$, where $(n)_{2}$ denotes the binary digit expansion of $n\in {\mathbb {N}}_{0}$ and $1(01^{*}0)^{*}10^{*}1$ has to be considered as a regular expression.

In this article, we are interested in the number of unbordered factors of length *n* in *t*; cf. [36, A309894]. We let *f* denote this sequence. The first few elements of this sequence are given in Table 7.

**Table 7:** First few elements of the sequence *f* which counts the number of unbordered factors in the Thue–Morse sequence of length *n*

*n*	0	1	2	3	4	5	6	7	8	9	10	11	12	13	14	15	...	23
*f*(*n*)	1	2	2	4	2	4	6	0	4	4	4	4	12	0	4	4	...	8

Theorem L characterizes the numbers *n* with $f(n) = 0$. However, for our purpose, this is not satisfying since we are interested in the numbers *f*(*n*) themselves. In particular, we hope that *f* is regular and that we can determine a linear representation for it, which consequently can be used to derive an asymptotic result for the summatory function of *f*.

Finally, before we come to the determination of a linear representation of *f*, the authors in [17] also investigated the growth rate of *f*, which is given as follows.

#### Theorem M

(Goč, Mousavi and Shallit  [17,  Theorem 2]) The inequality $f(n) \le n$ holds for all $n\ge 4$, with $f(n) = n$ infinitely often. This implies

$$\begin{aligned} \limsup _{n\ge 1}\frac{f(n)}{n} = 1. \end{aligned}$$

### Regularity and a Linear Representation

Goč, Mousavi and Shallit came up with the following recurrence relations, which will help us to conclude that *f* is 2-regular and to construct a 2-linear representation of *f*.

#### Theorem N

(Goč, Mousavi and Shallit  [17,  Proof of Theorem 2]) For the number *f*(*n*) of unbordered factors of length *n* in the Thue–Morse sequence, we have

$$\begin{aligned} f(4n)&= 2f(2n),&(n\ge 2)\\ f(4n + 1)&= f(2n + 1),&(n\ge 0)\\ f(8n + 2)&= f(2n + 1) + f(4n + 3),&(n\ge 1)\\ f(8n + 3)&= -f(2n + 1) + f(4n + 2),&(n\ge 2)\\ f(8n + 6)&= -f(2n + 1) + f(4n + 2) + f(4n + 3),&(n\ge 2)\\ f(8n + 7)&= 2f(2n + 1) + f(4n + 3).&(n\ge 3) \end{aligned}$$

This directly yields the following corollary.

#### Corollary O

For the number *f*(*n*) of unbordered factors of length *n* in the Thue–Morse sequence, we have

$$\begin{aligned} f(8n)&= 2f(4n),&(n\ge 1)\\ f(8n + 1)&= f(4n + 1),&(n\ge 0)\\ f(8n + 2)&= f(4n + 1) + f(4n + 3),&(n\ge 1)\\ f(8n + 3)&= -f(4n + 1) + f(4n + 2),&(n\ge 2)\\ f(8n + 4)&= 2f(4n + 2),&(n\ge 1)\\ f(8n + 5)&= f(4n + 3),&(n\ge 0)\\ f(8n + 6)&= -f(4n + 1) + f(4n + 2) + f(4n + 3),&(n\ge 2)\\ f(8n + 7)&= 2f(4n + 1) + f(4n + 3).&(n\ge 3) \end{aligned}$$

In particular, *f* is 2-recursive with offset $n_{0}=3$, exponents $M=3$ and $m = 2$ and index shift bounds $\ell = 0$ and $u=3$.

Together with the initial values given in Table 5, the recurrence relations stated in the previous corollary completely describe the sequence *f*.

In order to obtain a 2-linear representation of *f*, we first of all use Theorem E (instead of Theorem A which would lead to a linear representation of larger dimension). This will give us matrices $A_{0}$ and $A_{1}$ as well as a vector-valued sequence *v* such that the relations $v(2n) = A_{0}v(n)$ and $v(2n + 1) = A_{1}v(n)$ hold for all $n\ge 3$, i.e., a 2-linear representation with offset 3 of *f*.

With the notation of Theorem E, the matrices $B_{0}$ and $B_{1}$ are given by7.1where the relevant spectrum—relevant with respect to Proposition 4.7—isand all eigenvalues are simple.

We now apply Theorem E and obtain

$$\begin{aligned} v = \begin{pmatrix} f\\ f\circ (n\mapsto 2n)\\ f\circ (n\mapsto 2n+1)\\ f\circ (n\mapsto 4n)\\ f\circ (n\mapsto 4n+1)\\ f\circ (n\mapsto 4n+2)\\ f\circ (n\mapsto 4n+3) \end{pmatrix} \end{aligned}$$

as well as the matrices7.2Next, we use Theorem B in order to “adjust” the initial values of the recurrence relations of *f* given by Corollary O for $0\le n \le 2$.

For this purpose we recall the notation $\delta _{k}:{\mathbb {N}}_{0}\rightarrow {\mathbb {C}}$ with $\delta _{k}(n)~{{:=}}~ {\llbracket n=k \rrbracket }$ for $0\le k \le 2$ as introduced in Theorem B and set

$$\begin{aligned} w_{r,k} ~{{:=}}~ v(2k+r) - A_{r}v(k) \end{aligned}$$

for $0\le k \le 2$ and $0 \le r < 2$. Then the matrices $W_{0} = (w_{0,0},w_{0,1},w_{0,2})$ and $W_{1} = (w_{1,0},w_{1,1},w_{1,2})$ are given by

$$\begin{aligned} W_{0} = \begin{pmatrix} 0 &{}\quad 0 &{}\quad 0\\ 0 &{}\quad 0 &{}\quad 0\\ 0 &{}\quad 0 &{}\quad 0\\ -1 &{}\quad 0 &{}\quad 0\\ 0 &{}\quad 0 &{}\quad 0\\ -4 &{}\quad 0 &{}\quad 0\\ 4 &{}\quad 2 &{}\quad 0 \end{pmatrix} \text {and} ~~W_{1} = \begin{pmatrix} 0 &{}\quad 0 &{}\quad 0\\ 0 &{}\quad 0 &{}\quad 0\\ 0 &{}\quad 0 &{}\quad 0\\ -2 &{}\quad 0 &{}\quad 0\\ 0 &{}\quad 0 &{}\quad 0\\ 2 &{}\quad 2 &{}\quad 0\\ -8 &{}\quad -4 &{}\quad -4 \end{pmatrix}. \end{aligned}$$

Moreover, recall that we have introduced matrices $J_{0}$ and $J_{1}$ in (3.9) by

$$\begin{aligned} J_{r} ~{{:=}} ~\bigl ({\llbracket 2j = k - r \rrbracket }\bigr )_{\begin{array}{c} 0\le k< n_{0}\\ 0\le j < n_{0} \end{array}}, \end{aligned}$$

which in this example gives

$$\begin{aligned} J_{0} = \begin{pmatrix} 1 &{}\quad 0 &{}\quad 0\\ 0 &{}\quad 0 &{}\quad 0\\ 0 &{}\quad 1 &{}\quad 0\\ \end{pmatrix} \text { and}\; J_{1} = \begin{pmatrix} 0 &{}\quad 0 &{}\quad 0\\ 1 &{}\quad 0 &{}\quad 0\\ 0 &{}\quad 0 &{}\quad 0\\ \end{pmatrix}. \end{aligned}$$

Then by Theorem B we obtain a 2-linear representation $({\widetilde{A}}_{0}, {\widetilde{A}}_{1}, {\widetilde{v}})$ of *f* by the vector ${\widetilde{v}}$ given in block form as

$$\begin{aligned} {\widetilde{v}} = \begin{pmatrix} v\\ \delta _{0}\\ \delta _{1}\\ \delta _{2} \end{pmatrix} \end{aligned}$$

as well as the block matrices

7.3 $$\begin{aligned} {\widetilde{A}}_{0} = \begin{pmatrix} A_{0} &{}\quad W_{0}\\ 0 &{}\quad J_{0} \end{pmatrix} \text { and}\; {\widetilde{A}}_{1} = \begin{pmatrix} A_{1} &{}\quad W_{1}\\ 0 &{}\quad J_{1} \end{pmatrix}. \end{aligned}$$

This final 2-linear representation has dimension 10. By applying the minimization algorithm mentioned in Remark 3.6, we see that the smallest possible 2-linear representation of *f* has dimension 8.

### Joint Spectral Radius

Next, we determine the joint spectral radius of $B_{0}$ and $B_{1}$, which we need to determine the asymptotics of the summatory function of *f* in the next section.

#### Lemma 7.3

The joint spectral radius of ${\mathcal {B}}=\{B_{0}, B_{1}\}$ is 2 and ${\mathcal {B}}$ has the simple growth property.

#### Proof

As usual, $\Vert {\,\cdot \,} \Vert _\infty$ denotes the maximum norm of a vector and the row sum norm of a matrix. Let

$$\begin{aligned} T~{{:=}}~\text { diag}(2, 1/2, 1, 1) \end{aligned}$$

and consider the vector norm $\Vert {\,\cdot \,} \Vert _T$ defined by $\Vert {v} \Vert _T~{{:=}} ~\Vert {T^{-1}v} \Vert _\infty$. This vector norm induces the matrix norm $\Vert {\,\cdot \,} \Vert _T$ defined by $\Vert {G} \Vert _T~{{:=}} ~\Vert {T^{-1}GT} \Vert _\infty$.

We consider all products of two matrices in ${\mathcal {B}}$ and get

$$\begin{aligned} T^{-1}B_{0}^{2}T&= \begin{pmatrix} 4 &{}\quad 0 &{}\quad 0 &{}\quad 0 \\ 0 &{}\quad 1 &{}\quad 0 &{}\quad 0 \\ 0 &{}\quad 0 &{}\quad 1 &{}\quad 0 \\ 0 &{}\quad 0 &{}\quad 0 &{}\quad 1 \end{pmatrix},&T^{-1}B_{0}B_{1}T&= \begin{pmatrix} 0 &{}\quad 0 &{}\quad 2 &{}\quad 0 \\ 0 &{}\quad 0 &{}\quad 0 &{}\quad 2 \\ 0 &{}\quad 1 &{}\quad 0 &{}\quad 2 \\ 0 &{}\quad -\frac{1}{2} &{} \quad 1 &{}\quad 0 \end{pmatrix},\\ T^{-1}B_{1}B_{0}T&= \begin{pmatrix} 0 &{}\quad \frac{1}{2} &{} \quad 0 &{}\quad 1 \\ 0 &{}\quad -1 &{} \quad 2 &{} \quad 0 \\ 0 &{}\quad -\frac{1}{2} &{}\quad 1 &{}\quad 1 \\ 0 &{}\quad \frac{1}{2} &{}\quad 1 &{}\quad 0 \end{pmatrix},&T^{-1}B_{1}^{2}T&= \begin{pmatrix} 0 &{}\quad -\frac{1}{2} &{}\quad 1 &{}\quad 1 \\ 0 &{}\quad 2 &{} \quad 0 &{}\quad 2 \\ 0 &{}\quad \frac{1}{2} &{}\quad 1 &{}\quad 1 \\ 0 &{}\quad 1 &{}\quad 0 &{}\quad 3 \end{pmatrix}. \end{aligned}$$

We observe that all these matrices have row sum norm at most 4, which implies that

$$\begin{aligned}\max \{\Vert {G_1G_2} \Vert _T\mid G_1, G_2\in {\mathcal {B}}\}=4\end{aligned}$$

and therefore $\rho ({\mathcal {B}})\le 2$. Furthermore, we observe that $\Vert {B_0^k} \Vert _T=2^k$ holds for all even positive integers *k*. We conclude that $\rho ({\mathcal {B}})=2$ and that ${\mathcal {B}}$ has the finiteness property and thus the simple growth property. $\square$

#### Remark 7.4

As announced in Remark 4.2, the finiteness property may depend on the chosen norm. Indeed, the set ${\mathcal {B}}$ in Lemma 7.3 has the finiteness property with respect to the norm considered in the proof of Lemma 7.3, but does not have the finiteness property with respect to the row sum norm: By computing the eigendecomposition of $B_1$ or by induction on *k*, we obtain that the last row of $B_1^{k}$ is

$$\begin{aligned} \Bigl ( 0,\ \frac{2}{3}(2^k-(-1)^k),\ 0,\ \frac{1}{3}(2\cdot 2^k+(-1)^k) \Bigr ) \end{aligned}$$

for $k\ge 0$. In particular,

$$\begin{aligned} \Vert {B_1^k} \Vert _\infty \ge \frac{4}{3}2^k - \frac{1}{3}(-1)^k > 2^k \end{aligned}$$

holds for all $k\ge 1$.

### Asymptotics

Let $\eta \ge 1$ be an integer. We define the Dirichlet series

$$\begin{aligned} {\mathcal {F}}_{j}(s) ~{{:=}} ~\sum _{n\ge \eta }\frac{f(4n + j)}{(4n + j)^{s}} \end{aligned}$$

for $0\le j\le 3$ and set

$$\begin{aligned} {\mathcal {F}}(s) ~{{:=}}~ \begin{pmatrix} {\mathcal {F}}_{0}(s)\\ {\mathcal {F}}_{1}(s)\\ {\mathcal {F}}_{2}(s)\\ {\mathcal {F}}_{3}(s) \end{pmatrix}. \end{aligned}$$

In the following theorem, we state the main result of this section: We give an asymptotic formula for $F(N) ~{{:=}} ~\sum _{0\le n < N}f(n)$.

#### Theorem P

(Asymptotics for the number of unbordered factors) The summatory function *F* of the number *f*(*n*) of unbordered factors of length *n* in the Thue–Morse sequence satisfiesas $N \rightarrow \infty$, where $\kappa = \log _{2}(1+\sqrt{3}) = 1.44998431347650\ldots$ and $\Phi _{F}$ is a 1-periodic continuous function which is Hölder continuous with any exponent smaller than $\kappa - 1$. The Fourier coefficients of $\Phi _{F}$ can be computed efficiently.

Furthermore, the Dirichlet series ${\mathcal {F}}$ satisfies the functional equation

7.4 $$\begin{aligned} \bigl (I - 2^{-s}(B_{0} + B_{1})\bigr ){\mathcal {F}}(s) = \begin{pmatrix} {\mathcal {G}}_{0}(s)\\ {\mathcal {G}}_{1}(s)\\ {\mathcal {G}}_{2}(s)\\ {\mathcal {G}}_{3}(s) \end{pmatrix} \end{aligned}$$

for $\Re s > 1$, where $B_{0}$ and $B_{1}$ are the matrices given in (7.1), andwhere $(c_{j,k})_{0\le j<8, 0\le k\le 3}$ are the coefficients of the 2-recursive sequence in Corollary O. Moreover, ${\mathcal {G}}_{j}(s)$ is analytic for $\Re s > 1$ and $0\le j \le 3$, and, in particular, ${\mathcal {F}}(s)$ is meromorphic for $\Re s > 1$ and can only have poles $s = \log _{2}(\sqrt{3}+1) + \frac{2\pi i\mu }{\log 2}$ with $\mu \in {\mathbb {Z}}$.

**Fig. 4:**
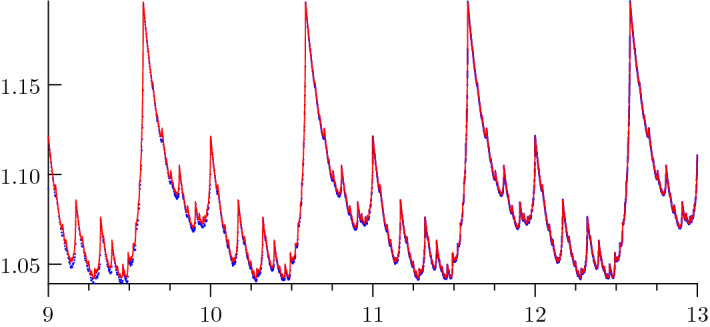
Fluctuation in the main term of the asymptotic expansion of the summatory function *F*. The plot shows the periodic fluctuation $\Phi _{F}(u)$ approximated by its Fourier series of degree 2000 (red) as well as the function $F(2^{u})/2^{\kappa u}$ (blue)

Table 8 and Fig. 4 show the first few Fourier coefficients and a plot of the periodic fluctuation of Theorem P, respectively.

**Table 8:** First few Fourier coefficients of $\Phi _{F}$

$\mu$	$\varphi _{\mu }$
0	1.081200224751780
1	$-0.0012296808157996 + 0.0157152473714320i$
2	$-0.0013742386970566 - 0.0110033266904103i$
3	$0.0083338522036749 + 0.0034850861320328i$
4	$-0.0042230458157050 - 0.0017461310727764i$
5	$0.0064055951023042 - 0.0018152583716649i$
6	$0.0004060978191922 + 0.0003312870598610i$
7	$0.0000421719576760 - 0.0039180258068148i$
8	$-0.0001216226961034 + 0.0017930566948364i$
9	$-0.0010960361908015 - 0.0012530549651823i$
10	$0.0024882524350784 - 0.0009385940552233i$

#### Proof of Theorem P

We use Theorem F with the linear representation $({\widetilde{A}}_{0},{\widetilde{A}}_{1},{\widetilde{v}})$ of *f* as obtained in Sect. 7.2. For this purpose, we work out the parameters needed in the theorem.

*Joint Spectral Radius.* Due to Lemma 7.3, Proposition 4.8 and Proposition 4.6, the joint spectral radius of ${\widetilde{A}}_{0}$ and ${\widetilde{A}}_{1}$ is 2. Moreover, the simple growth property holds. So we set $R = 2$.

*Eigenvalues.* Let ${\widetilde{C}} = {\widetilde{A}}_{0} + {\widetilde{A}}_{1}$. We could of course directly calculate the eigenvalues of ${\widetilde{C}}$. However, we want to emphasize the relation to the matrices $B_{0}$ and $B_{1}$ as given in (7.1) and thus, to the recurrence relations given by the property that the sequence *f* is 2-recursive. Let $A_{0}$ and $A_{1}$ be the matrices in (7.2) and set $C {{:=}} A_{0} + A_{1}$. Then applying Proposition 4.7 yieldsas well as $m_{C}(\lambda ) = m_{B_{0} + B_{1}}(\lambda )$ for $\lambda \in \sigma (B_{0}+B_{1})$, and we recall thatsee Sect. 7.2. Moreover, all eigenvalues of $B_{0} + B_{1}$ are simple and thus, we have $m_{C}(\lambda ) = 1$ for all $\lambda \in \sigma (B_{0}+B_{1})$.

Due to Proposition 4.4, we haveand $m_{{\widetilde{C}}}(\lambda ) = m_{C}(\lambda )$ for . All in all, this means for the spectrum of ${\widetilde{C}}$ thatSo the only relevant eigenvalues of ${\widetilde{C}}$, i.e., the only eigenvalues $\lambda$ with $|\lambda | \ge R$, are $\sqrt{3}+1$ with $m_{{\widetilde{C}}}(\sqrt{3}+1) = 1$ and 2 with $m_{{\widetilde{C}}}(2) = 1$.

Now applying Theorem F yields the result for the asymptotic expansion. Finally, Proposition 4.9 implies the correctness of the functional equation as stated in (7.4). $\square$

## Proofs

### Proofs of the Reductions to *q*-Regular Sequences in the General Case

For the proof of Theorem A, we need the following lemma.

#### Lemma 8.1

Let $q\ge 2$, $M > m \ge 0$ and $\ell \le u$ be integers, and let $\ell '$ and $u'$ be as defined in (3.2). Furthermore, let *d* be an integer with $\ell '\le d\le q^{M-1}-q^{m}+u'$ and write $d = d'q^{M} + r'$ with $0\le r' < q^{M}$ and $d'\in {\mathbb {Z}}$. Then we have

8.1 $$\begin{aligned} \ell ' \le q^{m}d' + \ell \text { and} ~q^{m}d' + u \le u'. \end{aligned}$$

If additionally $r' \ge q^{M-1}$ holds, then

8.2 $$\begin{aligned} q^{m}d' + q^{m} + u \le u'. \end{aligned}$$

#### Proof

First of all, we note that the equalities

8.3 $$\begin{aligned} {\left\lfloor \frac{{\left\lfloor x\right\rfloor } + a}{b}\right\rfloor } = {\left\lfloor \frac{x + a}{b}\right\rfloor } \text { and} ~{\left\lceil \frac{{\left\lceil x\right\rceil } + a}{b}\right\rceil } = {\left\lceil \frac{x + a}{b}\right\rceil } \end{aligned}$$

hold for all $x\in {\mathbb {R}}$ and *a*, $b\in {\mathbb {Z}}$ with $b > 0$; see Graham, Knuth and Patashnik [19,  p. 72].

We now show that the left inequality of (8.1) is true. If $\ell \ge 0$, then $\ell ' = 0$ and the inequality follows. If $\ell < 0$, then we havewhich is equivalent to $\ell ' \le q^{m}d' + \ell$.

For the remaining two inequalities, we distinguish between negative and non-negative upper bounds *u*.

1. $u \le -1$. This implies $u' = q^{m} - 1$ and as a consequence, we have $d \le q^{M-1} - 1$ as well as $d' \le 0$. The right inequality of (8.1) as well as (8.2) follow directly.
2. $u \ge 0$. This implies $u' = q^{m} - 1 + {\left\lceil \frac{uq^{M-m}}{q^{M-m}-1}\right\rceil }$, and we have 8.4 This is equivalent to $q^{m}d' + u \le u'$, and (8.1) is shown. Now let $r'\ge q^{M-1}$, then it follows that
  $$\begin{aligned} q^{m}d' + q^{m} + u&= \frac{d - r'}{q^{M-m}} + q^{m} + u\\&\le \frac{u' + q^{M - 1} - q^{m} - q^{M-1} + q^{M} + uq^{M-m}}{q^{M-m}}\\&= \frac{u' + q^{M} - q^{m} + uq^{M-m}}{q^{M-m}}. \end{aligned}$$
  We can now use the same steps as above (from (8.4) on) and obtain $q^{m}d' + q^{m} + u \le u'$, which shows that (8.2) holds. This completes the proof.

$$\square $$

#### Proof of Theorem A

Let *v* be as stated in Theorem A and let $(c_{r,k})_{0\le r<q^M, \ell \le k\le u}$ be the coefficients of the *q*-recursive sequence *x*. We have to show that there are matrices $A_{0}$, $\ldots$, $A_{q-1}$ such that $v(qn + r) = A_{r}v(n)$ holds for all $n\ge n_{1}$ and $0\le r < q$, as required in the definition of regular sequences. This is equivalent to the property that each component of $v(qn + r)$ is a linear combination of the entries in *v*(*n*), where all sequences are restricted to $n\ge n_{1}$.

We fix $0\le r < q$ and split the proof into three parts depending on the indices of the blocks $v_{j}$ of *v* as introduced in (3.3). In each part, the choice of the components of *v* as defined in (3.4a) and (3.4b) allows to represent $v_{j}(qn+r)$ as a linear combination of the entries of *v*.

Furthermore, we let $\text {ind}_{v}(x\circ (n\mapsto q^{j}n+d))$ denote the absolute position of some sequence $x\circ (n\mapsto q^{j}n+d)$ in the vector *v*, absolute in the sense that we disregard the block form of *v*.^12^ Moreover, we write $v_{j,d}$ for the sequence $x\circ (n\mapsto q^{j}n+d)$ where this notation is convenient.

**Part 1.** At first, we consider blocks $v_{j}$ of *v* with $0\le j < m$.

*Components of* $v_{j}$: Let $0\le d < q^{j}$ and consider the component $v_{j,d} = x\circ (n\mapsto q^{j}n + d)$ of *v*. Then we get

$$\begin{aligned} v_{j,d}(qn+r)&= x\bigl (q^{j}(qn + r) + d\bigr )\\&= x(q^{j+1}n + q^{j}r + d). \end{aligned}$$

We claim that $x\circ (n\mapsto q^{j+1}n+q^{j}r + d)$ is a component of $v_{j+1}$. To show this, we first observe that

8.5 $$\begin{aligned} 0\le q^{j}r + d \le q^{j}(q-1) + q^{j} - 1 = q^{j + 1} - 1. \end{aligned}$$

For $j \le m-2$, this immediately proves the claim. If $j=m-1$, then the estimates $\ell '\le 0$ (see Remark 3.6) and $q^{j+1}-1 = q^{m}-1 \le u'$ together with (8.5) ensure the validity of the claim.

*Corresponding Rows of* $A_{r}$: The previous considerations imply that the row $\text {ind}_{v}(v_{j,d})$ of $A_{r}$ has zeros in every entry except one. Specifically, the entry in column $\text {ind}_{v}\bigl (v_{j+1, q^{j}r + d}\bigr )$ is 1.

**Part 2.** Secondly, we consider blocks $v_{j}$ of *v* with $m\le j \le M-2$.

*Components of* $v_{j}$: Let $\ell '\le d \le q^{j} - q^{m} + u'$. Then the sequence $x\circ (n\mapsto q^{j}n + d)$ is a component of $v_{j}$ and it holds that

$$\begin{aligned} x\bigl (q^{j}(qn+r) + d\bigr ) = x(q^{j+1}n + q^{j}r + d) \end{aligned}$$

with

$$\begin{aligned} \ell '\le d \le q^{j}r + d \le q^{j}(q - 1) + q^{j} - q^{m} + u' = q^{j+1} - q^{m} + u'. \end{aligned}$$

This implies that the sequence $x\circ (n\mapsto q^{j}(qn + r) + d)$ is a component of *v*, namely in the block $v_{j+1}$.

*Corresponding Rows of* $A_{r}$: Also in this part, the row $\text {ind}_{v}(v_{j,d})$ of $A_{r}$ consists of zeros except for position $\text {ind}_{v}(v_{j+1,q^{j}r + d})$ where we have a 1.

**Part 3.** Finally, we have a look at the last block $v_{M-1}$ of *v*.

*Components of* $v_{M-1}$: Let $\ell ' \le d \le q^{M-1} - q^{m} + u'$ and consider the component $v_{M-1,d} = x\circ (n\mapsto q^{M-1}n + d)$ of $v_{M-1}$. Write $d=d'q^{M} + r'$ with $0\le r' \le q^{M}-1$ and $d'\in {\mathbb {Z}}$. The component of $v(qn+r)$ which corresponds to $v_{M-1,d}$ is given by

$$\begin{aligned} x\bigl (q^{M-1}(qn+r) + d\bigr ) = x\bigl (q^{M}(n+d') + q^{M-1}r + r'\bigr ) = x\bigl (q^{M}(n + d') + \tilde{r}\bigr ) \end{aligned}$$

with $\tilde{r} {{:=}} q^{M-1}r + r'$. Note that $\tilde{r}\le q^{M-1}(q-1)+q^{M}-1=2q^M-q^{M-1}-1$. We distinguish the following two cases with respect to the parameter *r* for determining the rows of $A_r$ corresponding to the block $v_{M-1}$.

1. $0\le \tilde{r} < q^{M}$. We have 8.6 for $n + d'\ge n_{0}$ due to (3.1). As $d'={\left\lfloor d/q^{M}\right\rfloor }\ge {\left\lfloor \ell '/q^{M}\right\rfloor }$ holds, the condition $n + d'\ge n_{0}$ is fulfilled for $n\ge n_{1}$ by definition of $n_{1}$. We need to show that $\ell ' \le q^{m}d' + k \le u'$ holds for all $\ell \le k\le u$. We apply Lemma 8.1 and directly obtain both inequalities, i.e., $\ell ' \le q^{m}d' + k$ and $q^{m}d' + k \le u'$, by (8.1). This implies that the sequence $x\circ (n\mapsto q^{M-1}(qn + r) + d)$ is a linear combination of sequences in *v*, where all sequences are restricted to $n\ge n_{1}$. *Corresponding rows of* $A_{r}$: We have a look at row $\text {ind}_{v}(v_{M-1,d})$ of $A_{r}$. The first couple of entries are zero, up to position $\text {ind}_{v}(v_{m,q^{m}d'+\ell }) - 1$. On position $\text {ind}_{v}(v_{m,q^{m}d'+\ell })$ up to position $\text {ind}_{v}(v_{m,q^{m}d'+u})$ we have the entries $c_{\tilde{r},\ell }$, ..., $c_{\tilde{r},u}$, followed by zeros up to the end of the row.
2. $q^{M} \le \tilde{r} < 2q^{M} - q^{M-1} -1$. This implies $r' \ge q^{M-1}$ and $0\le \tilde{r} - q^M < q^M$. We obtain 8.7 for $n \ge n_{1} - 1$, again due to (3.1). Thus, we need to argue that $\ell '\le q^{m}d' + q^{m} + k \le u'$ holds for all $\ell \le k\le u$. For this purpose, we again use Lemma 8.1. The inequality $q^{m}d' + q^{m} + k \le u'$ directly follows from (8.2). For the lower bound we obtain  This shows that also in this case, the sequence $x\circ (n\mapsto q^{M-1}(qn + r) + d)$ is a linear combination of sequences in *v*, where again all sequences are restricted to $n\ge n_{1}$. *Corresponding rows of* $A_{r}$: We again have a look at row $\text {ind}_{v}(v_{M-1,d})$ of $A_{r}$. The first couple of entries are zero, up to position $\text {ind}_{v}(v_{m,q^{m}d'+q^{m}+\ell }) - 1$. On position $\text {ind}_{v}(v_{m,q^{m}d'+q^{m}+\ell })$ up to position $\text {ind}_{v}(v_{m,q^{m}d'+q^{m}+u})$ we have the entries $c_{\tilde{r}-q^M,\ell }$, ..., $c_{\tilde{r}-q^M,u}$, followed by zeros up to the end of the row.

**Finalizing the Proof.** At this point, we have completed constructing matrices $A_r$ for $0\le r < q$ row by row and have shown that $(A_0, \ldots , A_{r-1}, v)$ is a linear representation of *x*. We now gather the information on $A_r$ which was discussed in the proof. We let *D* denote the dimension of $A_r$. If $a_{i}$ is the *i*th row of $A_{r}$, define $j\in {\mathbb {N}}_0$ and $d\in {\mathbb {Z}}$ by $i=\text {ind}_{v}(v_{j, d})$. Then we have

8.8 $$\begin{aligned} a_{i}^{\top } = {\left\{ \begin{array}{ll} \bigl ({\llbracket k = \text {ind}_{v}(v_{j+1,q^{j}r+d}) \rrbracket }\bigr )_{1\le k \le D} &{} \text {if } 0\le j \le M-2,\\ \bigl (c_{\tilde{r},k + k_{1}}\cdot {\llbracket \ell \le k + k_{1} \le u \rrbracket }\bigr )_{1\le k \le D} &{} \text {if } j = M-1 \text { and } \tilde{r} < q^{M},\\ \bigl (c_{\tilde{r}-q^M,k + k_{2}}\cdot {\llbracket \ell \le k + k_{2} \le u \rrbracket }\bigr )_{1\le k \le D} &{} \text {if } j = M-1 \text { and } \tilde{r} \ge q^{M}, \end{array}\right. }\qquad \end{aligned}$$

where

- $$\tilde{r} ~{{:=}}~ q^{M-1}r + r'$$ with $$d = d'q^{M} + r'$$, $$d'\in {\mathbb {Z}}$$ and $$0\le r' < q^{M}$$,
- $$k_{1} ~{{:=}} ~\ell -\text {ind}_{v}(v_{m,q^{m}d'+\ell })$$ and
- $$k_{2} ~{{:=}} ~\ell -\text {ind}_{v}(v_{m,q^{m}d'+q^{m}+\ell })$$.

Note that all these parameters depend on *i*. $\square$

#### Proof of Theorem B

The definitions of $\delta _{k}$ and $w_{r,k}$ in Theorem B imply thatholds for all $0\le r < q$ and $n \ge 0$. We further havefor all $0\le k < n_{0}$, $0\le r < q$ and $n\ge 0$. With ${\widetilde{v}}$ and ${\widetilde{A}}_{r}$ as defined in (3.7) and (3.8), it follows that ${\widetilde{v}}(qn + r) = {\widetilde{A}}_{r}{\widetilde{v}}(n)$ holds for all $0\le r < q$ and $n\ge 0$. Consequently, $({\widetilde{A}}_{0}, \ldots , {\widetilde{A}}_{q-1},{\widetilde{v}})$ is a *q*-linear representation of *x* and thus, *x* is *q*-regular. The statement about the shape of $J_r$ follows from the definition of $J_r$. This completes the proof of the theorem. $\square$

Corollary C directly follows by combining Theorem A and Theorem B.

#### Proof of Corollary D

First of all, note that for a *q*-regular sequence *g*, its shifted version $g\circ (n\mapsto n + d)$ is *q*-regular for all integers $d\in {\mathbb {Z}}$; see Allouche and Shallit [1,  Theorem 2.6 and the subsequent remark].

We use the notation introduced in Theorem A. In order to prove the corollary, we construct a *q*-linear representation with offset $n_{1}$ of *x* along the lines of the proof of Theorem A: Parts 1 and 2 of the proof can be adopted unchanged, but we have to carefully adapt Part 3: In (8.6) and (8.7), we have to apply (3.10) instead of (3.1) and obtain8.9in the first case and8.10in the second case, where $n\ge n_{1}$ and ${\left\lfloor \ell '/q^{M}\right\rfloor }\le d' \le {\left\lfloor (q^{M-1} - q^{m} + u')/q^{M}\right\rfloor }$. Thus, if we append the vectors of the *q*-linear representations of the *q*-regular sequences $g_{s}\circ (n\mapsto n + d'')$ for $0\le s < q^{M}$ and ${\left\lfloor \ell '/q^{M}\right\rfloor }\le d'' \le {\left\lfloor (q^{M-1} - q^{m} + u')/q^{M}\right\rfloor } + 1$ to the vector *v* as given by (3.3) and analogously join the corresponding matrices together—with some additional entries 1 caused by $g_{\tilde{r}}(n+d')$ and $g_{\tilde{r}-q^{M}}(n+d'+1)$ in (8.9) and (8.10), respectively—then we obtain a *q*-linear representation with offset $n_{1}$ of *x*.

Finally, we correct the offset of the *q*-linear representation by applying Theorem B. Consequently, *x* is *q*-regular. $\square$

### Proof of the Reduction to *q*-Regular Sequences in the Special Case

#### Proof of Theorem E

We split the proof into two parts depending on the indices of the blocks of *v*.

**Part 1.** At first, we consider blocks $v_{j}$ with $0\le j<m$. These blocks coincide with (3.4a) in Theorem A. Moreover, these blocks correspond to the first $\sum _{0\le j <m}q^{j} = (q^{m} - 1)/(q-1)$ rows of the matrices $A_{0}$, ..., $A_{q-1}$ and thus, also to the rows of $J_{r0}$ and $J_{r1}$ for $0\le r < q$. By the proof of Theorem A, each of these rows consists of zeros and exactly one 1, which in particular means that the only entries of $J_{r0}$ and $J_{r1}$ are zeros and ones as stated in the theorem. It remains to show that the matrices $J_{r0}$ are upper triangular with zeros on the diagonal. This follows from the fact that

$$\begin{aligned} \text {ind}_{v}\bigl (x\circ (n\mapsto q^{j}(qn + r) + d)\bigr ) > \text {ind}_{v}\bigl (x\circ (n\mapsto q^{j}n + d)\bigr ) \end{aligned}$$

holds for all $0\le j < m$, $0\le r < q$ and $0\le d < q^{j}$.

**Part 2.** We take a look at the block $v_{m}$. The definition of $B_{r}$ in (3.11) implies that

$$\begin{aligned} v_{m}(qn + r) = B_{r}v_{m}(n) \end{aligned}$$

holds for all $0\!\!\le \!\! r\!\! < \!\!q$, which, together with Part 1, directly implies that $(A_{0}, \dots , A_{q-1}, v)$ as given in (3.12) and (3.14) is indeed a linear representation of *x*. $\square$

### Proofs of the Spectral Results

We start with the proof of Proposition 4.4.

#### Proof of Proposition 4.4

Due to (3.8), we have

$$\begin{aligned} {\widetilde{C}} = \begin{pmatrix} C &{}\quad W\\ 0 &{}\quad J \end{pmatrix} \end{aligned}$$

with the $n_{0}\times n_{0}$ matrix $J = \sum _{0\le r < q}J_{r}$ and $W = \sum _{0\le r < q}W_{r}$, and thus, $\sigma ({\widetilde{C}}) = \sigma (C)\cup \sigma (J)$. By the properties of $J_r$ noted in Theorem B, *J* is a lower triangular matrix with $\text { diag}(J) = (1,0,\dots ,0)$. This yields . Furthermore, we can say the following. If $n_{0} = 1$, then $J=(1)$ and thus, . If $n_{0} \ge 2$, then $\text { diag}(J) = (1,0,\dots ,0)$ holds with $n_{0}- 1 \ge 1$ zeros which implies . This yields the result as stated. $\square$

Before proving Lemma 4.5, we need another lemma.

#### Lemma 8.2

Let ${\mathcal {G}}$ be a finite set of $(D_1+D_2)\times (D_1+D_2)$ block upper triangular matrices. For $G\in {\mathcal {G}}$ write

$$\begin{aligned} G = \begin{pmatrix} G^{(11)}&{} G^{(12)}\\ 0&{}G^{(22)} \end{pmatrix} \end{aligned}$$

where the block $G^{(ij)}$ is a $D_i\times D_j$ matrix for $1\le i\le j\le 2$.

Let $R_1$ and $R_2$ be non-negative constants such that

$$\begin{aligned} \Vert {G^{(ii)}_1\ldots G^{(ii)}_k} \Vert =O(R_i^k) \end{aligned}$$

holds for all $G_1$, ..., $G_k\in {\mathcal {G}}$, $i\in \{1, 2\}$ and $k\rightarrow \infty$.

Assume that $R_1\ne R_2$ and set $R{{:=}} \max \{R_1, R_2\}$. Then

$$\begin{aligned} \Vert {G_1\ldots G_k} \Vert =O(R^k) \end{aligned}$$

holds for all $G_1$, ..., $G_k\in {\mathcal {G}}$ and $k\rightarrow \infty$.

#### Proof

As both the assumption and the statement of the lemma do not depend on the particular norm by the norm equivalence theorem, it suffices to consider the maximum norm on vectors and thus the row sum norm on matrices.

It is easily shown by induction on *k* that

$$\begin{aligned} G_1\ldots G_k = \begin{pmatrix} G^{(11)}_1\ldots G^{(11)}_k &{} \sum _{j=1}^{k}\bigl (G^{(11)}_1\ldots G^{(11)}_{j-1}\bigr ) G^{(12)}_j \bigl (G^{(22)}_{j+1}\ldots G^{(22)}_k\bigr )\\ 0 &{} G^{(22)}_1\ldots G^{(22)}_k \end{pmatrix}. \end{aligned}$$

If one of $R_1$, $R_2$ equals zero, all except at most one of the upper right summands vanish and the result follows easily.

Otherwise, assume without loss of generality that $R_1<R_2$. Then

$$\begin{aligned} \Vert {\sum _{j=1}^{k}\bigl (G^{(11)}_1\ldots G^{(11)}_{j-1}\bigr ) G^{(12)}_j \bigl (G^{(22)}_{j+1}\ldots G^{(22)}_k\bigr )} \Vert&= O\biggl (\sum _{j=1}^{k} R_1^{j-1}R_2^{k-j}\biggr )\\ {}&= O\biggl (R_2^{k} \sum _{j=1}^{k} \Bigl (\frac{R_1}{R_2}\Bigr )^j\biggr ) =O(R_2^{k}) \end{aligned}$$

as $k \rightarrow \infty$, because the last sum can be estimated from above by a bounded geometric series.

Adding the contributions of the rows leads to the result. $\square$

We are now able to prove Lemma 4.5.

#### Proof of Lemma 4.5

As the joint spectral radius as well as the simple growth property do not depend on the choice of the norm, we only consider the row sum norm in this proof.

By definition of the joint spectral radius, for every choice of constants $R_i>\rho ({\mathcal {G}}^{(i)})$,

$$\begin{aligned} \Vert {G^{(ii)}_1\ldots G^{(ii)}_k} \Vert =O(R_i^k) \end{aligned}$$

holds for $G_1$, ..., $G_k\in {\mathcal {G}}$, $1\le i\le s$ and $k\rightarrow \infty$ (where the implicit constant depends on the $R_i$). If ${\mathcal {G}}^{(i)}$ has the simple growth property, we may also choose $R_i=\rho ({\mathcal {G}}^{(i)})$. Without loss of generality, we can choose the $R_i$ to be pairwise distinct. Then repeated application of Lemma 8.2 shows that

8.11 $$\begin{aligned} \Vert {G_1\ldots G_k} \Vert = O((\max _{1\le i\le s}R_i)^k) \end{aligned}$$

holds for all $G_1$, ..., $G_k\in {\mathcal {G}}$ and $k\rightarrow \infty$. Consequently, we obtain $\rho ({\mathcal {G}})\le \max _{1\le i\le s}R_i$. Taking the infimum over all possible choices of $R_i$ shows that $\rho ({\mathcal {G}})\le \max _{1\le i\le s}\rho ({\mathcal {G}}^{(i)})$. As the diagonal blocks of the product of block triangular matrices are the products of the corresponding diagonal blocks, it follows that $\rho ({\mathcal {G}})\ge \max _{1\le i\le s}\rho ({\mathcal {G}}^{(i)})$.

If there is a unique $i_0\in \{1, \ldots , s\}$ such that $\rho ({\mathcal {G}}^{(i_0)})=\rho ({\mathcal {G}})$ and ${\mathcal {G}}^{(i_0)}$ has the simple growth property, we can choose $R_{i_0}=\rho ({\mathcal {G}}^{(i_0)})$ and $R_i<\rho ({\mathcal {G}}^{(i_0)})$ for $i\ne i_0$ and the simple growth property of ${\mathcal {G}}$ follows from (8.11). $\square$

#### Proof of Proposition 4.6

As the matrices $J_r$ are lower triangular matrices with diagonal elements 0 and 1, Lemma 4.5 (or Jungers [25,  Proposition 1.5]) implies that $\rho ({\mathcal {J}})\le 1$. As $J_0$ has a one on the diagonal, we actually have $\rho ({\mathcal {J}})=1$.

Since all matrices ${\widetilde{A}}_{0}$, ..., ${\widetilde{A}}_{q-1}$ are upper triangular block matrices, we can apply Lemma 4.5 (or Jungers [25,  Proposition 1.5]) once more to obtain the first part of (4.5). The “in particular”-statement follows directly from (4.5).

Finally, Lemma 4.5 shows that $\widetilde{{\mathcal {A}}}$ has the simple growth property under the assumptions stated in the last part of Proposition 4.6. $\square$

#### Proof of Proposition 4.7

Observe that we have

$$\begin{aligned} C = \begin{pmatrix} J_{00} + \cdots + J_{(q-1)0} &{} J_{01} + \cdots + J_{(q-1)1}\\ 0 &{} B_{0} + \cdots + B_{q-1} \end{pmatrix}. \end{aligned}$$

So *C* is an upper triangular block matrix, which implies

$$\begin{aligned} \sigma (C) = \sigma (J_{00} + \cdots + J_{(q-1)0}) \cup \sigma (B_{0} + \cdots + B_{q-1}). \end{aligned}$$

By Theorem E, $J_{00} + \cdots + J_{(q-1)0}$ is a triangular matrix with zero diagonal, so its only eigenvalue is 0, which yields the result. $\square$

#### Proof of Proposition 4.8

The statement $\rho ({\mathcal {J}}) = 0$ follows by Lemma 4.5 and the fact that every matrix in ${\mathcal {J}}$ is an upper triangular matrix with zeros on the diagonal. The rest of the proposition can be proven in analogy to Proposition 4.6. $\square$

### Proof of the Functional Equation in the Special Case

#### Proof of Proposition 4.9

For $0\le j < q^{m}$ and $\Re s>\log _q \rho +1$, replacing *n* by $qn+\mu$ yieldsWe now use (3.1) and obtain

$$\begin{aligned} {\mathcal {X}}_{j}(s)&= \sum _{\mu =0}^{q-1}\;\sum _{n\ge \eta }\frac{\sum _{k=0}^{q^{m}-1}c_{\mu q^{m} + j,k}\,x(q^{m}n + k)}{(q^{m+1}n + \mu q^{m} + j)^{s}} + \sigma \\&= q^{-s}\sum _{\mu =0}^{q-1}\;\sum _{k=0}^{q^{m}-1}c_{\mu q^{m} + j,k}\sum _{n\ge \eta }\frac{x(q^{m}n + k)}{\bigl (q^{m}n + \frac{\mu q^{m} + j}{q}\bigr )^{s}} + \sigma \\&= q^{-s}\sum _{k=0}^{q^{m}-1}\Bigl (\sum _{\mu =0}^{q-1}c_{\mu q^{m} + j,k}\Bigr ){\mathcal {X}}_{k}(s) + {\mathcal {Y}}_{j}(s), \end{aligned}$$

where ${\mathcal {Y}}_{j}(s)$ is given by

8.12 $$\begin{aligned} {\mathcal {Y}}_{j}(s) = q^{-s}\sum _{k=0}^{q^{m}-1}\;\sum _{\mu =0}^{q-1}c_{\mu q^{m}+j,k}\biggl (\sum _{n\ge \eta }\frac{x(q^{m}n + k)}{\bigl (q^{m}n + \frac{\mu q^{m} + j}{q}\bigr )^{s}} - {\mathcal {X}}_{k}(s)\biggr ) + \sigma . \end{aligned}$$

Note that $\sum _{k=0}^{q^{m}-1}\sum _{\mu =0}^{q-1}c_{\mu q^{m} + j,k}{\mathcal {X}}_{k}(s)$ is the *j*th row of $(B_0+\cdots +B_{q-1}){\mathcal {X}}(s)$. It is easy to see that

$$\begin{aligned} |\frac{\mu q^{m} + j}{q} - k| < q^{m} \le q^{m}\eta + k \end{aligned}$$

holds for all $0\le \mu < q$, $0\le j < q^{m}$ and $0\le k < q^{m}$. Consequently, we apply [20,  Lemma 6.3] with $n_{0} = q^{m}\eta + k$ (see (4.6)) as well as $\beta = \frac{\mu q^{m}+j}{q} - k$ and obtain

8.13 $$\begin{aligned} \sum _{n\ge \eta }\frac{x(q^{m}n + k)}{\bigl (q^{m}n + \frac{\mu q^{m} + j}{q}\bigr )^{s}} - {\mathcal {X}}_{k}(s) = \sum _{n \ge 1}\left( {\begin{array}{c}-s\\ n\end{array}}\right) \Bigl (\frac{\mu q^{m} + j}{q} - k\Bigr )^{n}{\mathcal {X}}_{k}(s + n), \end{aligned}$$

again for all $0\le \mu < q$, $0\le j < q^{m}$ and $0\le k < q^{m}$. Furthermore, the right-hand side of (8.13) is analytic for $\Re s > \log _{q}\rho$. Plugging (8.13) into (8.12) and reordering terms yields the result. The functional equation (4.7) implies that ${\mathcal {X}}_j(s)$ is meromorphic for $\Re s>\log _q\rho$ and can only have poles where $q^{s}\in \sigma (B_{0} + \cdots + B_{q-1})$. $\square$

## Footnotes

1 Throughout this paper, we let ${\mathbb {N}}$ denote the set of positive integers and write . Moreover, we write sequences in functional notation, i.e., we write a (complex-valued) sequence *x* as a function $x:{\mathbb {N}}_{0}\rightarrow {\mathbb {C}}$, and as a consequence, the *n*th element of the sequence is denoted by *x*(*n*). In addition, we consider a vector of complex-valued sequences $v = (x_{1}, \dots , x_{D})^{\top }$ as a vector-valued function $v:{\mathbb {N}}_{0}\rightarrow {\mathbb {C}}^{D}$, and its evaluation is defined component-wise, i.e., $v(n) = (x_{1}(n),\dots ,x_{D}(n))^{\top }$.

2 By the definition of *q*-regular sequences as given in Sect. 2, when representing every subsequence of the form $h(2^jn+r)$ as a linear combination of the same finite number of sequences, this needs to hold for all $n\ge 0$. In particular, this needs to hold for $n = 0$, which is not fulfilled in the example. However, this is only a minor technical issue and can be fixed by adding some appropriate sequence to the linear combination. Details about this repair are provided in Theorem B.

3 In the standard literature [1, 2], these sequences are called *k*-regular instead of *q*-regular.

4 The results given in Sects. 3.2 and 3.3 are valid for sequences $x:{\mathbb {N}}_{0}\rightarrow R$, where *R* is a commutative Noetherian ring. However, in places where we speak about the asymptotics of a sequence, we always consider complex-valued sequences.

5 While the binary sum of digits *s* does not directly fit into the framework with $M=1$ and $m=0$, it is a 2-recursive sequence with exponents $M = 2$ and $m = 1$, since the recurrence relations $s(4n)=s(2n)$, $s(4n + 1)=s(2n + 1)$, $s(4n + 2)=s(2n + 1)$ and $s(4n + 3)= -s(2n) + 2s(2n+1)$ follow from (2.2) for all $n\ge 0$. This means that in this and similar cases we can get rid of the inhomogeneity by increasing the exponents.

6 We use Iverson’s convention: For a statement *S*, we set ${\llbracket S \rrbracket } = 1$ if *S* is true and 0 otherwise; see also Graham, Knuth and Patashnik [19, p. 24].

7 We set $x(n)=0$ for $n < 0$ to ensure that all blocks are well-defined. By the choice of $n_1$, this does not have any factual consequences.

8 We set $x(n)=0$ for $n < 0$ to ensure that all vectors are defined. Other definitions of values for negative arguments are possible, but would result in other values for $W_{r}$.

9 We let $\{ z \} {{:=}} z - {\left\lfloor z\right\rfloor }$ denote the fractional part of a real number *z*.

10 Note that this sequence has a strong connection to Stern–Brocot trees, which were first discovered independently by Stern [35] and Brocot [7]. This is the reason that Stern’s diatomic sequence is also known as *Stern–Brocot sequence.*

11 Strictly speaking, $d(0) = 0$ follows from (5.1b) for $n = 0$.

12 We assume that the operator $\text {ind}_{v}$ is “intelligent” enough to recognize the sequence $x\circ (n\mapsto q^{j}n+d)$ in *v* by the parameters *j* and *d*, even if there are other sequences in *v* that are equal to $x\circ (n\mapsto q^{j}n+d)$ (in the sense that all elements of the sequences are equal). For reasons of simplicity and readability, we refrain from develop this more explicitly at this point.
